# Defect-induced monopole injection and manipulation in artificial spin ice

**DOI:** 10.1038/s41467-022-31309-0

**Published:** 2022-06-25

**Authors:** Robert Puttock, Ingrid M. Andersen, Christophe Gatel, Bumsu Park, Mark C. Rosamond, Etienne Snoeck, Olga Kazakova

**Affiliations:** 1 Quantum Materials and Sensors, National Physical Laboratory, Teddington, UK; 2 Centre d’Elaboration de Materiaux et d’Etudes Structurales, Toulouse, France; 3 School of Electronic and Electrical Engineering, University of Leeds, Leeds, UK

**Keywords:** Magnetic devices, Magnetic properties and materials

## Abstract

Lithographically defined arrays of nanomagnets are well placed for application in areas such as probabilistic computing or reconfigurable magnonics due to their emergent collective dynamics and writable magnetic order. Among them are artificial spin ice (ASI), which are arrays of binary in-plane macrospins exhibiting geometric frustration at the vertex interfaces. Macrospin flips in the arrays create topologically protected magnetic charges, or emergent monopoles, which are bound to an antimonopole to conserve charge. In the absence of controllable pinning, it is difficult to manipulate individual monopoles in the array without also influencing other monopole excitations or the counter-monopole charge. Here, we tailor the local magnetic order of a classic ASI lattice by introducing a ferromagnetic defect with shape anisotropy into the array. This creates monopole injection sites at nucleation fields below the critical lattice switching field. Once formed, the high energy monopoles are fixed to the defect site and may controllably propagate through the lattice under stimulation. Defect programing of bound monopoles within the array allows fine control of the pathways of inverted macrospins. Such control is a necessary prerequisite for the realization of functional devices, e. g. reconfigurable waveguide in nanomagnonic applications.

## Abstract (web summary)

Artificial spin ice systems offer a promising platform to study the motion of emergent magnetic monopoles, but controlled nucleation of monopoles is challenging. Here the authors demonstrate controlled injection and propagation of emergent monopoles in an artificial spin ice utilizing ferromagnetic defects.

## Introduction

Artificial spin ice (ASI) are two-dimensional arrays of single domain magnetic nanoelements, which act as Ising macrospins and constitute a geometrically frustrated lattice such that the energies at each vertex are never simultaneously satisfied^1–3^. ASI were first introduced as projections of spin ice crystals, which consist of pyrochloric tetrahedral lattices corner-linked by rare-earth ions^4,5^. Due to the large intrinsic crystal field acting on the rare earth ions they demonstrate idealistic Ising spin pointing towards or away from each unit cell center, resulting in a system exhibiting geometric frustration and residual entropy.

It is possible to uniquely tailor the designs of ASI and explore a plethora of frustration physics that would be more challenging in 3D material systems; including phase transitions^6–10^ and collective dynamics^11–14^. The 2D nature, modularity, and room temperature operation of ASI also makes them versatile in application in reconfigurable magnonics^15–19^, magnetricity^20–22^, and novel computation^23–26^.

**Fig. 1:**
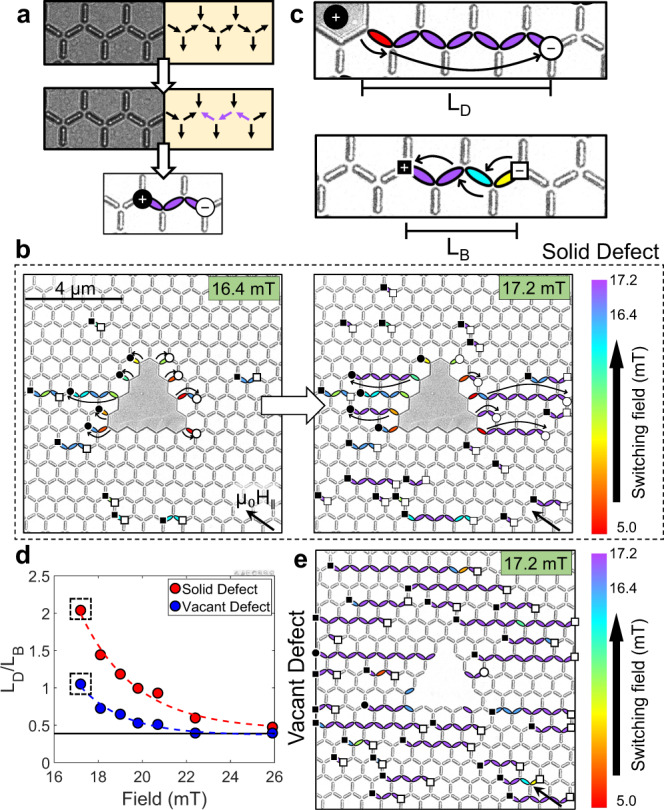
Monopole nucleation in DASI. **a** Under-focused LTEM micrograph (left sub-panel) and schematics (right sub-panel) of Kagomé vertex junctions before and after monopole-antimonopole (MP-AMP) nucleation (top and center panels, respectively); bottom panel, an example of labeled LTEM where switched macrospins are represented by ellipses whose color shows their respective switching field; MP-AMP charges are drawn for reference. **b** Labeled LTEM image at in-plane fields μ_0_H_‖_ = 16.4 mT and μ_0_H_‖_ = 17.2 mT (left and right panels, respectively) of the solid DASI undergoing field reversal; MPs that nucleate at the defect-ASI interface are represented as circles, whereas those that form spontaneously are represented by squares; curved arrows represent the defect-bound MP propagation direction. **c** Labeled LTEM image with representations of defect-dependent and independent MP propagation (top and bottom panels, respectively), where the respective Dirac string lengths (L_D_ and L_B_) are indicated. **d** Mean L_D_/L_B_ ratio at increasing field magnitude for the solid DASI and a lattice with a vacant defect **e**. Lattices depicted in b (right panel) and e are indicated in d by the boxes.

One exciting subfield of both spin ice and ASI research is investigating the properties of emergent monopole-antimonopole (MP-AMP) pairs^27–29^. These are oppositely charged magnetic charge states, bound through a string of inverted dipoles (Dirac string, DS), are topologically protected from the inability to annihilate one component of the pair without annihilating the other^3,29,30^ (Fig. 1a). Upon formation, the MP-AMP pairs propagate under excitation through the lattice as it undergoes magnetization reversal. However, it is challenging to controllably nucleate individual MP-AMP pairs below the switching field of the lattice without relying on discontinuities at the boundaries or uncontrollable extrinsic factors from the fabrication process^31^. Nucleation pads have been previously used to controllably inject MPs into connected lattices at the array edges^32^ but pinning often impedes propagation of MPs without modification of the frustrated junctions^26,33^. In addition, both the charge and countercharge are free, meaning they can propagate under excitation. Tailorable and controllable MP dynamics is paramount to ASI realizing its technological potential.

Introducing defects into structured lattices disrupts the local energy landscape, which in turn can have bountiful effects on the underlying physics. For example, defects in solid-state systems have applications as single photon point-sources in quantum information processing due to highly localized excitons bound to the defect-site^34,35^. Defects have also been utilized previously in ASI, such as induced strain in a square ASI lattice by Burgers vector  distortion as presented by Drisko et al.^36^. The lattice dislocation creates distinctive domain boundaries under thermal agitation from an inability to form ground-state order. Such strain-based defects in ASI provide additional insight into dislocations in spin ice and other 3D frustrated materials.

Replacing non-magnetic sites with ferromagnetic defects in ASI can be used to mimic the behaviors of, e.g., stuffed spin ice crystals^37^. Substituting non-magnetic atoms with rare earth ions in the spin ice lattice has been shown to increase monopole density at the substitution sites. The defects act as both nucleation centers and as pinning sites, which in turn impacts the monopole dynamics^38–40^. Additionally, oxygen vacancies in Yb_2_Ti_2_O_7-δ_ have an analogous effect by inducing magnetism on the Ti-sites^39^.

In the present work, a defect-ASI (DASI) lattice is introduced that uses dipolar ferromagnetic coupling to investigate individual MP dynamics. The disconnected Kagomé lattice was chosen as it possesses a large ground-state degeneracy and equivalent interaction strengths between NIs at the frustrated junctions^41^. A solid triangular ferromagnetic defect was fabricated at one site in the center of the lattice in the same lithography step. The defect replaces six primitive cells of the Kagomé lattice and mimics the shape of nitrogen vacancies in hexagonal boron nitride^35^. The defect possesses honeycomb edges to mitigate lattice distortions such as those introduced by the Burgers vector. As such, neighboring vertex junctions are highly sensitive to the focused defect stray-field at these locations resulting in MP nucleation sites in the local vicinity^42–44^.

## Results

In Fig. 1, Lorentz transmission electron microscopy (LTEM) was used to investigate the reversal mechanism of the DASI lattice. Images were acquired at each ~1 mT iteration of in-plane component of the applied field (μ_0_H_‖_). The bright/dark intensity in the LTEM image within the nanostructures arise from the deflection of the electron beam when passing through a magnetic material by the Lorentz force. A nanostructure that has undergone a binary switch will present a reversed contrast. Image segmentation tools were used to ascertain the switching fields of individual NIs by measuring changes in their intensity across the field sequence. Colored ellipses were superimposed onto the LTEM micrographs to represent the individual NI switching fields as shown in the schematic (Fig. 1a, bottom panel), and the brightness and intensity has been modified to highlight the lattice topography.

Cascaded magnetization reversal in NIs trace the charge-conserving DSs that bind the MP-AMP pair. Labeled LTEM micrographs of the DASI lattice at μ_0_H_‖_ = 16.4 mT and 17.2 mT are displayed in Fig. 1b (left and right panels, respectively). Black and white symbols represent the MP charges (positive and negative, respectively), where circles represent MPs that nucleated from the defect-lattice interface. MP-AMP pairs that form in the lattice bulk are instead represented by squares (Fig. 1c).

There are three notable observations in Fig. 1b (left panel):

1. A higher concentration of MPs adjacent to the defect is the result of lower nucleation fields relative to the rest of the lattice, see the red-orange-yellow ellipses.
2. The separation of MPs of opposite charge either side of the defect.
3. Each MP formed at the defect-site is bound through a DS to the defect rather than an AMP countercharge, meaning the ratio of MPs to AMPs is no longer equivalent.

Observation #1 reveals that the defect is a nucleation site for emergent MPs. The reduced switching field in defect-adjacent NIs is the result of the high-stray-field of the defect when saturated, which triggers the magnetization reversal in neighboring NIs. The magnetic configuration of the saturated defect creates surface charges at the defect-lattice interface (Fig. 1c, top panel) relative to the field vector. The defect inherently stabilizes the defect-bound MPs upon nucleation by effectively encompassing the AMP counter-pair. Thus, the defect behaves as a universal source and sink for charge conservation in the lattice resulting in non-bijective MP charges in the lattice.

Figure 1b (right panel) shows the labeled LTEM micrograph one iteration further along the field sweep (μ_0_H_‖_ = 17.2 mT). Purple ellipses represent all changes in magnetization between images in Fig. 1b. Here, many of the defect-bound MPs have propagated further from the defect into the lattice, extending the DS length. This is in addition to the nucleation of some MP-AMP pairs in the lattice bulk as μ_0_H_‖_ approaches the lattice intrinsic switching field.

The ratio of the mean length of the defect-bound DSs (${L}_{{{{{{\rm{D}}}}}}}$, Fig. 1c, top panel) to the mean-length of the DSs that form in the lattice bulk (${L}_{{{{{{\rm{B}}}}}}}$, bottom panel) was calculated and plotted for each experimental image at 17.2 mT ≤ μ_0_H_‖_ ≤ 25.9 mT (Fig. 1d). The limit imposed by the image field-of-view (FOV) is also plotted (solid black line). The solid DASI (red circles) was compared to a similar lattice with a vacant defect site (blue circles, and Fig. 1e) to identify nucleation events that result from the presence of a solid defect instead of a lattice discontinuity. Supplementary Movies 1–6 show the magnetic reversal of both lattices, and analyzed images are presented in Supplementary Figs. 1–3.

The vacant defect lattice exhibits ${L}_{{{{{{\rm{D}}}}}}}$/${L}_{{{{{{\rm{B}}}}}}}$ ≈ 1.1 when μ_0_H_‖_ = 17.2 mT and decreases exponentially upon higher field application to the limit imposed by the FOV. This value evidences that the vacant defect does not influence the lattice reversal mechanism any more than the usual extrinsic factors (such as edge conditions). The lattice with solid defect also follows an exponential trend as field is increased, but the expansion of the defect-bound DSs dominate at field magnitudes below 20 mT, where the ratio exceeds ${L}_{{{{{{\rm{D}}}}}}}$/${L}_{{{{{{\rm{B}}}}}}}$ >> 1.0. Above 20 mT, the majority of NIs along this propagation direction have flipped as the lattice switching field is reached, forming a striped DS phase^29^. As such, the quantity of defect-bound MPs is dwarfed by surrounding excitations. This reveals that low-field operation is necessary to maximize upon the defect-bound MP dynamics.

The stray-field of the saturated defect is the impetus for MP injection at low-fields into the lattice compared to discontinuities from lattice edges or the vacant defect. The defect induced reversal in neighboring NIs results in MPs that appear to propagate easier through the lattice at lower fields before the critical lattice switching field is reached. However, in this arbitrary field orientation, some of the defect bound MPs are also subject to pinning close to the defect. This is in line with previous works where nucleation pads were fabricated at the edge of the array. We focus on overcoming the pinning conditions later in the manuscript using the properties of the defect design.

**Fig. 2:**
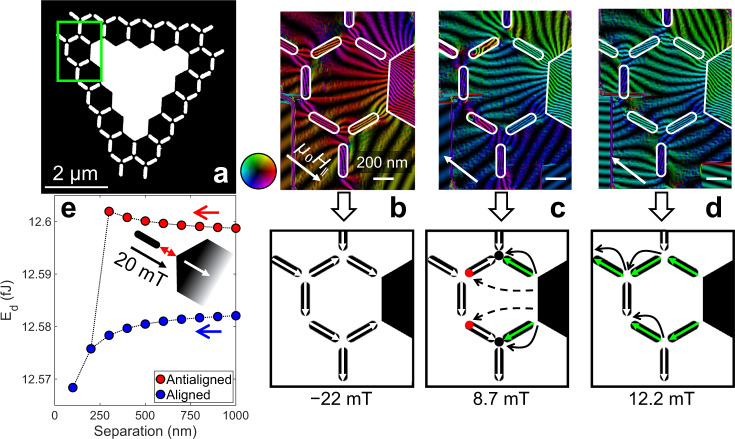
Visualization of flux-coupling in DASI. **a** Schematic of a smaller DASI lattice with analyzed region represented by green rectangle. **b**–**d** electron holograph reconstructed phase contour images and schematics (top and bottom panels, respectively) at snapshots of field reversal in DASI along the direction of the white arrow; colors and contour density represent the direction and magnitude of the magnetic flux, respectively, with reference to the color-wheel. Solid black arrows on schematics indicate the MP propagation direction, whereas dashed arrows indicate the long-range defect-vertex coupling that stabilizes the vortex domain walls (red dots). White and green arrows represent the initial and switched island magnetization, respectively. **e** Modeled demagnetization energy (*E*_d_) of the saturated defect and a single island initially magnetized anti-parallel (red) and parallel (blue) to the applied field (μ_0_H_‖_ = 20 mT) as a function of defect-island separation.

To understand how the defect aids the nucleation and propagation processes the LTEM results have been complemented with off-axis electron holography (EH), which maps the magnetic induction normal to the electron beam^45^. To achieve an adequate FOV, a much smaller lattice was investigated (Fig. 2a).

Figure 2b–d displays the magnetic phase contour images extracted from EH and related schematics representing the NI magnetic configuration. Figure 2b shows the initial magnetic state at µ_0_H_|| _= − 22 mT. Here, the magnetization of the NIs lie along their respective easy axes but possess a summed net component along the field direction. The defect is fully saturated along the field direction because of its weaker shape anisotropy compared to the NIs. The flux-line density of the phase contour images is an indirect quantification of the flux-strength and magnetic coupling. In Fig. 2b, the defect strongly couples with the nearest NI that is oriented parallel to µ_0_H_||_. The three frustrated junctions sampled were in equivalent energy-states, which is reflected by the similar inter-island flux density. The background contrast shows the defect has a far-reaching stray-field that also weakly couples to far-away NIs. This is a first indication that the defect not only couples with nearest islands but also interacts with at least the next-nearest neighboring vertices as well.

Figure 2c captures magnetization reversal in the lattice at µ_0_H_|| _= 8.7 mT. The magnetization of the defect has completely reversed from the initial state along the new field direction. This has triggered the closest NIs to flip and nucleate MPs in the neighboring vertex junctions. The phase images also capture the nucleation of vortex domain walls in the next-nearest NI apices, which signals the start of the subsequent propagation step. Under increased field µ_0_H_|| _= 12.2 mT (Fig. 2d), the defect-bound MPs have propagated to the edge of the lattice where they have terminated.

The direction of the NI magnetization reversal in Fig. 2c is initially counterintuitive as it would be expected that the domain wall would nucleate at the site of the most frustrated junction (i.e. the three-in configuration). Instead, the reversal begins at the opposite vertex. In Fig. 2c, the flux-lines show the defect coupling with the vortex domain wall under reversal to enable this anomalous reversal and aid the initial propagation step.

Micromagnetic modeling^46,47^ was performed to understand the defect-NI coupling length-scales. Fig. 2e plots the demagnetization energy (*E*_d_) of a single NI and defect system as a function of separation distance (*r*) (*inset*). The red and blue plots show the energies when the initial NI magnetization is aligned antiparallel and parallel to the saturated defect (set by the application of the field µ_0_H_|| _= 20 mT), respectively. Here the NI magnetization reverses when *r* < 300 nm. The defect-NI interaction drops off with the expected *r*^3^ proportionality with minimal interaction beyond 500 nm.

From the results in Fig. 2, it is conclusive that the defect has a pivotal role in MP nucleation, and a role in the propagation mechanism (at least in the initial stages). This stray-field induced reversal is thus separate from that caused by lattice edge-states, which does not demonstrate this reversal behavior. Extra modeling of the interaction between one NI and the defect in different positions surrounding the defect are included in Supplementary Fig. 4. It shows that the defect shape anisotropy results in an asymmetry in NI-defect coupling under saturation. This is because of a weaker stray-field at the triangular base of the defect compared to the apex, relative to the field direction.

**Fig. 3:**
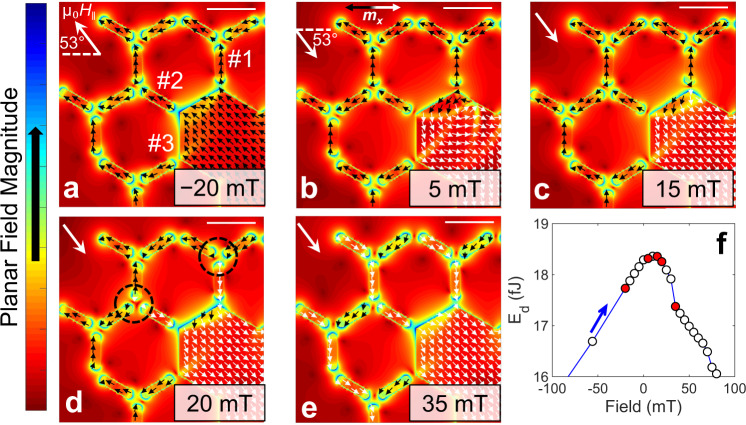
Micromagnetic modeling of monopole injection. **a**–**e** 2D planar field magnitude colormaps of the modeled lattice (top-left corner of the lattice is displayed for direct comparison with Fig. 2) at in-plane field (µ_0_H_||_) iterations: −20 mT; 5 mT; 15 mT; 20 mT; and 35 mT, respectively, along the indicated direction (top-left corner). Arrows represent the discretized magnetization vector colored to represent its alignment with respect to *x*-axis (as shown in b, top-right). Scale-bars represent 500 nm. **f** Plot of the demagnetization energy (*E*_d_) as a function of µ_0_H_||_; blue arrow shows the sweep-direction and red circles represent the energies of the field-maps in **a**–**e**.

To aid the interpretation of the EH results, micromagnetic modeling was performed on the full lattice depicted in Fig. 2a. Figure 3a–e presents the discretized planar field-magnitude vector of the top-left corner of the structure at a planar applied field angle θ = 53° from the *x*-axis, and arrows represent the discretized in-plane magnetization vectors. The field magnitude directly represents the dipolar coupling strength of the lattice components at the vertex junctions and the defect-NI interfaces. Fig. 3f plots the *E*_d_ of the full lattice as a function of µ_0_H_||_, where the states depicted in Fig. 3a-e are indicated by red circles. Simulations of the full lattice are available in Supplementary Fig. 5 and Supplementary Movie 7.

Fig. 3b, c reveals that the *E*_d_ maxima is a result from the defect saturation under the Zeeman field opposite to the lattice magnetization. This high-energy magnetostatic interaction at the defect-NI interfaces explains why neighboring NIs #1–2 flip at a reduced µ_0_H_||_ in Fig. 3d. Defect-bound MPs are injected into the lattice at µ_0_H_|| _= 20 mT onto the junctions indicated (Fig. 3d, black dashed circles) and are shown to propagate to the edges of the lattice upon greater field application (Fig. 3e), which complements the EH results. Fig. 3 also shows that NI #3 very weakly interacts with the saturated defect due to a mismatch in spin alignment to the magnetization in the defect. As a result, NI #3 is far less affected by the reversal of the defect along this field direction, which ultimately governs the propagation axis.

The influence of shape anisotropy of the defect-ASI system is further studied through micromagnetic modeling. We look at three individual cases for direct comparison:

#1. Kagomé lattice with vacant defect site

#2. Isolated defect

#3. Defect-ASI lattice.

**Fig. 4:**
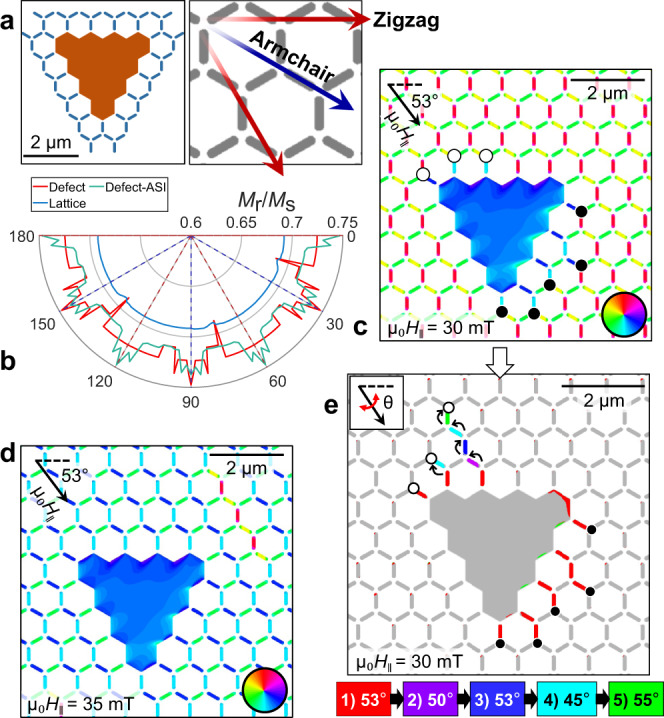
Modeling of defect-induced MP propagation. **a** (Left panel) Small DASI lattice where individual defect and lattice components (orange and blue, respectively) are combined to form Defect-ASI; (Right panel) Schematic of zigzag and armchair axes (burgundy and navy-blue, respectively). **b** Angular dependence of the normalized remanent magnetization (*M*_r_/*M*_s_) of the lattice (blue), defect (red) and combined Defect-ASI (green) systems achieved by application of μ_0_H_‖_ = 2 T saturation field then subsequently left to evolve at remanence; dashed burgundy and navy-blue lines represent zigzag and armchair axes, respectively. **c**, Modeled magnetization configuration (color represents angle of the magnetic vector with respect to the *x*-axis) of the DASI lattice at µ_0_H_|| _= 30 mT and *θ* = 53°, where nucleated monopoles are presented as black and white circles (positive and negative charges, respectively). **d**, Modeled magnetization map of the same lattice at µ_0_H_|| _= 35 mT and *θ* = 53°, where most of the NIs along the field-direction have switched. **e**, Differential map of modeled switching events, at µ_0_H_|| _= 30 mT, as θ is varied within the range 45–55°, NI flips are colored according to the legend (e.g., red NIs represent the switched states from **c**).

As we are interested primarily in the defect-lattice interface, we focus again on the smaller lattice displayed in full in Fig. 4a (left panel). The three systems are assessed for their easy- and hard-axis alignments by applying a saturating field (µ_0_H_|| _= 2 T) along a specified direction, θ, and then relaxing the system under zero-field. From this, the remanent magnetization (*M*_r_) is calculated for each system. Here, a larger *M*_r_ is representative of a relatively easy axis.

The traditional Kagomé lattice has six-fold symmetry and two identifiable axes of interest, as shown in Fig. 4a (right panel): the zigzag axis that runs perpendicular to 1/3 of NIs; and the armchair axis, which runs parallel to 1/3 of NIs (30° from the zigzag axis).

Fig. 4b is a polar-axis plot of the *M*_r_ for different saturating field angles (θ) for all three systems, where the zigzag and armchair axes are indicated by the dashed burgundy and navy-blue lines, respectively. The lattice without the ferromagnetic defect displays a largely flat *M*_r_ (blue line), which is indicative of the rigid shape anisotropy of the NIs. One clear hard-axis transition point occurs at, e.g., θ–45°, which lies between the armchair and zigzag axes and represents the reversal of some of the NIs in the lattice. From this, a similar hard-axis would be expected at, e.g., θ–15°, but a combination of the 2° step-size and non-Ising symmetry breaking at the frustrated junctions likely impeded this observation^48^. Comparative plots of *E*_d_ and relevant magnetization maps of the different systems are displayed in Supplementary Figs. 6, 7, respectively.

The polar plot for the defect is far less discrete because of its comparatively weaker shape anisotropy. There is clear easy-axis alignment when the field is applied down the defect symmetry axis, normal to the triangular base, as expected for a triangular nanostructure^49,50^. The honeycomb edges behave as additional domain wall pinning locations, which yields the spikes when the field is aligned with the arm-chair axes. A large metastable region exists when the field is applied ±15° from each zigzag axis. This coincides with the formation of a “buckle” domain configuration^50^ where a domain wall forms down the defect’s symmetry axis. The defect hard axes coincide with those described also for the Kagomé lattice.

The green-line presents the *M*_r_ of the combined defect-ASI lattice, which exhibits similarly to the isolated defect except the metastable buckle magnetization state does not preferentially form when the field is aligned along one of the armchair axes. This creates an additional easy-axis alignment because of the lattice-defect interaction at the interface under the field history. This indicates a strong lattice-defect interaction when the field is aligned along the armchair axis that is not present for the individual systems.

These insights are now used to explore defect-bound MP propagation in the lattice without incurring parasitic MP-AMP nucleation/propagation events in the wider lattice. Fig. 4c-e displays the modeled micromagnetic configuration for a larger array under specific applied field protocols. To investigate this, we now model the response of a larger lattice (described in Methods). Fig. 4c displays the initial state of the lattice after MP nucleation when μ_0_H_‖_ = 30 mT is applied at *θ* = 53°. Under greater field, μ_0_H_‖_ = 35 mT, the intrinsic switching field of the lattice is reached (Fig. 4d), where most NIs along the field direction have flipped and MPs have terminated at the lattice boundaries. This accurately represents the trends in the experimental results in Figs. 1, 2 but occurs in a much smaller field window because the simulations are performed in the absence of thermal noise.

An alternative route for controlling the propagation of defect-bound MPs is presented in Fig. 4e, which does not induce defect-independent nucleation. This is achieved by rotating the applied field in the sample plane. Fig. 4e is a differential image that colors the switched NIs at each sequential field-angle from the x-axis, θ, at fixed field magnitude. For example, red shading represents the reversed NIs from the starting configuration depicted in Fig. 4c, and purple shading represents subsequent switching events that result from canting the field to *θ* = 50°.

Further analyses of the effects of angle on the nucleation and propagation mechanisms are displayed in Supplementary Note 6 of the SI. We show through modeling that defect-bound monopole nucleation can be maximized when the field is applied directly down the zigzag axis. However, rotation of the field to an off-axis component is required to encourage favorable defect-bound MP propagation after initialization.

We have shown that the position of the defect-bound MPs can be controlled by sequentially teetering the field direction without nucleating MP-AMP pairs away from the defect. By varying θ, the vector alignment with respect to the easy axis of the individual NI is changed. This subtle change in the local energies at the MP locations induces controllable propagation of defect-bound MPs to overcome pinning without new MP-AMP pairs nucleating in the lattice.

**Fig. 5:**
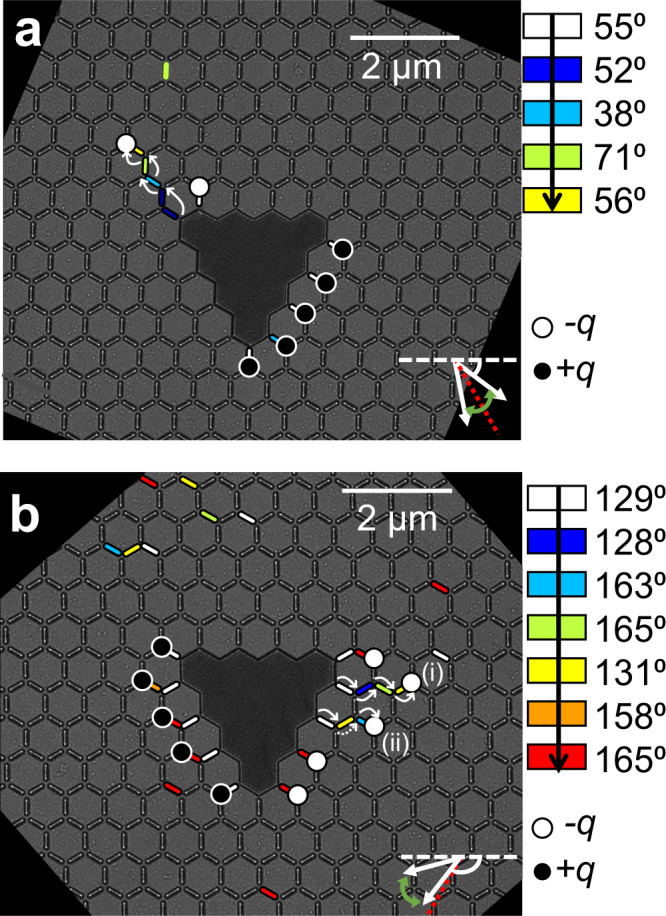
Defect-bound MP manipulation. **a**, **b** LTEM images of Defect ASI lattice as it undergoes the field protocol described by the color scale (µ_0_H_|| _= 21 mT). Colored islands represent NI switching relative to the color-bar provided, and final positions of defect-bound negative (white) and positive (black) MPs are indicated by circles. Solid and dashed white arrows indicate one-step and two-step MP propagation mechanisms, respectively; and dashed red-line indicates the closest zigzag axis to the net field direction (bottom-right of each image).

This method is now experimentally demonstrated. Fig. 5a, b presents LTEM images where the field is teetered both about the zigzag and armchair axes (*θ* = 60° and 150° from the lattice horizontal), respectively. Switched NIs throughout the field sequence are indicated by colored shading. In Fig. 5a, the process is initialized by saturating the lattice towards the top-left of the image. Subsequently, the field is ramped in the inverse direction (θ = 55°) until the first NIs at the defect-lattice interface switch (white islands). The sequential switching of NIs under the field protocol are shaded according to the color-scale. It is evident that the field protocol results in a controlled propagation of the negative monopole (white circle) at the top-left corner of the defect. Meanwhile, few switching events occur in the greater lattice and at the base of the defect.

In Fig. 5b, the field protocol is rotated 74° to that in Fig. 5a, so that the initial field angle θ = 129°. Here, the field is teetered about the armchair axis at 150° (the red-dashed line represents the zigzag axis at 120°). This is equivalent to the steps presented in Fig. 4e because of the rotational symmetry. The negative monopoles (i) and (ii) preferentially propagate from the defect throughout the field protocol until the final field step (red islands), where many NIs switch.

The preferred propagation response in the ASI system occurs offset from the apex of the triangular defect (relative to the field direction), which complements the modeling in Fig. 4e and in Supplementary Note 6. This observation is initially surprising as the field is teetering about the predetermined defect easy axis, which incurs a larger stray-field at the narrow end of the defect under saturation. However, the symmetry of the armchair axis prevents further propagation of this MP until the field angle deviates significantly. This process enables the propagation at the triangular defect edges rather than the corner.

MP (ii) in Fig. 5b propagates in a non-linear fashion as the center NI in the chain of three is the last to switch in the sequence (indicated by the dashed arrow). This repeatedly occurred at this location. We suspect it may be a consequence of the next-nearest neighbor coupling shown in Fig. 2, which could flip a different NI in the frustrated vertex depending on the field history. However, it is difficult to distinguish this from local variations in the NI switching field.

In both field orientations it was increasingly difficult to propagate the MP charge beyond 5–6 islands away from the defect without also incurring defect independent nucleation of MP-AMP pairs (see Supplementary Fig. 13). This represents a distance dependence where the stray-field of the defect assists MP diffusion. This evidences an even greater effect than anticipated from the results described in Fig. 2.

Limited defect-independent nucleation events also occurred in the bulk of the lattice in Fig. 5 and Supplementary Fig. 13, deviating from the idealized response in the modeling from Fig. 4. However, these extra nucleation events were rare and appeared to be less mobile under the field protocol.

We note that when the defect bound MP meets an MP-AMP pair an annihilation event^51^ occurs. The result of this annihilation is the formation of a defect bound monopole of the same charge, with a longer DS. Further evidence for this is shown in figure Supplementary Fig. 14. This provides a topological robustness as local MP-AMP pairs act to extend the defect-MP distance. Therefore, regardless of annihilation events, the defect will always be bound to the final monopole position.

In Fig. 5a, b, two of the negative MPs propagated throughout the field sequence (white circles), whereas the positive MPs (black circles) remain pinned, which mirrors the modeling from Fig. 4d. The applied field is misaligned from the nearest zigzag axis at 60° relative to the triangular symmetry axis. This field misalignment results in a focusing of the stray-field at the narrow apex of the defect. This creates an anisotropic effect on the NIs in close proximity to the defect from the stray field coupling. This asymmetric behavior results in a limited diode-like response from the pinning of similar MPs at the defect base. Further modeling that investigates varying field angle and magnitude are provided in S6 of the SI, which show that the pinning can be overcome with a wider field window.

## Discussion

We have demonstrated the effect and mechanism by which a ferromagnetic defect with shape anisotropy inserts MPs into the Kagomé lattice. The field-assisted athermal MP injection is shown to occur below the critical switching field of the lattice and have lasting effects on the MP propagation steps. These effects are distinct from those caused by traditional extrinsic factors and are susceptible to the defect shape anisotropy because of the direct coupling at the defect-lattice interface. It is reasonable to expect that other methods to manipulate the magnetic state could also utilize the defect-lattice coupling mechanism, e.g., by spin-polarized currents, microwave^19,52^ or thermal excitation^36,51,53–55^, or nanoscale field manipulation^56,57^.

Upon nucleating the defect-bound monopole we demonstrated, both experimentally and through modeling, a mechanism to manipulate specific defect-bound MPs with minor variations in the applied field direction at fixed magnitude to overcome initial pinning mechanisms. The combination of the defect design, its positioning in the lattice, and the experimental procedure permits the MP control. The modeling in Fig. 4, and in Supplementary Figs. 8–10, demonstrated how the variations in field history allow for controllable MP propagation without inducing unwanted nucleation events when the critical switching field of the lattice is not reached. The modelling agrees with the experimental results displayed in Fig. 5. Although limited defect-independent nucleation events occurred in the experimental realization, particularly when the field direction approached the armchair axis, nucleated MP-AMP pairs did not freely propagate as the defect-bound counterparts under the field conditions. This is attributed to the influence of the stray-field of the defect.

Below the critical lattice switching field the defect’s triangular shape anisotropy promoted greater mobility of one charge of defect-bound MPs over the other relative to the field history. This diodic behavior provides an extra degree of freedom when exploring distinct MP dynamics. In the case where the defect-bound MP does meet an MP-AMP pair, as seen in Fig. 5b, they annihilate to form a single defect-bound MP. These final points provide some robustness against potential crosstalk in applications requiring rewritable pathways between, e.g., a source and drain as is required for reconfigurable waveguides. In the present work, the same defect-lattice design has been investigated throughout the measurements; different defect designs or defect-lattice pairings may yield yet even further improvements on the intrinsic MP dynamics.

In summary, the inclusion of a solid defect into an otherwise continuous ASI lattice locally reduces the energy barrier required to create MPs in the local defect vicinity. These MPs are separated by charge as governed by the defect magnetization and conserved by remaining bound to the defect rather than an AMP countercharge. The weak shape anisotropy of the defect relative to field alignment is pivotal for low-energy MP nucleation and results in asymmetric charge mobility. The defect-bound MPs are shown to propagate below the lattices’ intrinsic switching field by teetering the field alignment with respect to the lattice axes, providing a means for greater control over individual MPs. Free MPs bound to a single location could be beneficial for applications that require greater dynamic control over the reversed pathway, such as functional magnetic networks for data transport and storage^58^ or magnetricity-based devices^22^.

## Methods

### Fabrication and dimensions

Patterned Py structures were fabricated on Si_3_N_4_ membranes (*t* = 20 nm). Membranes were spin-coated with ZEP520A (*t* = 140 nm) and baked at 180 °C. The ASI arrays were then patterned by *e*-beam lithography (100 kV, 343 µC/cm^2^) and developed in n-amyl acetate. Py (*t* = 20 nm, 2 Å/s) with a Pt capping layer to prevent oxidation (*t* = 2 nm, 1 Å/s) were evaporated. Finally, lift-off was performed in DMSO. The lattice is composed of elongated nanoislands (NIs) whose anisotropy forces their respective magnetization to lie in-plane along each respective long axis with the Ising 2^1^ degrees of freedom. The individual NIs in both lattices are 380 × 100 nm in size and the defect is 2.95 µm in length. The small array extends to just one or two frustrated vertices in the network away from the defect location, whereas the extended array spans approximately 50 × 50 µm^2^ area.

### Lorentz transmission electron microscopy

Lorentz TEM is the simplest TEM technique for observing magnetic domains. The electron beam experiences a Lorentz force when interacting with the magnetic induction of the sample, which deflects the transmitted e-beam dependent on the magnetization direction. In Fresnel mode, the image is defocused and the magnetically deflected e-beams of two neighboring magnetic domains of opposite magnetization create two bright or dark lines that indicate the in-plane direction of the magnetic domains.

The magnetization and switching processes were observed using Fresnel mode in LTEM in a Hitachi HF-3300 (I2TEM) microscope operated at 300 kV and fitted with a cold field-emission gun and image corrector (B-Corr). The observations were done using the normal sample stage of the I2TEM with the objective lens switched off. Magnetic switching studies were performed by applying an in situ magnetic field by controlling the current of the objective lens of the microscope. As the magnetic field generated by the lens is oriented perpendicular to the image plane, the sample was tilted out of the plane to apply external magnetic field components parallel to the sample plane (µ_0_H_||_). The sample was also rotated in its plane to adjust the direction of the applied field along different axes of the ASI network. µ_0_H_⊥_ is considered constant throughout the data sequence as the small tilt angles used result in a negligible variation in the out of plane component in the interesting tilt range of the experiment. For example, a sample tilt of 2° is required to achieve µ_0_H_|| _= 17.4 mT, which results in an out-of-plane field component µ_0_H_**⊥**_ = 487.7 mT. This is only a 300 µT difference from zero-tilt.

The magnetic switching presented in Fig. 1 was observed by acquiring defocused LTEM Fresnel images for different in plane field strengths. The magnetic field created by the objective lens was kept at constant 0.5 T at the object plane, while tilt sweep series were performed to acquire Fresnel mode LTEM images of the system. This was done by tilting the sample to +7° (i.e., applying µ_0_H_|| _= 60 mT) saturating the lattice magnetization to point in the in-plane field direction, before gradually tilting back to −2.4°, acquiring LTEM images at each 0.1° tilt increment. corresponding to a ~1 mT change of applied in-plane field per tilt increment in the range of the magnetic switching events.

The experimental MP propagation in Fig. 5 was performed by rotating the sample in plane inducing a rotation of µ_0_H_||_ on the ASI network. The lattice magnetization was first saturated by tilting the sample +7° (*i.e*., applying µ_0_H_|| _= 60 mT). The sample was then tilted in opposite direction until MPs nucleated at −2.4° (*i.e*., applying µ_0_H_|| _= 21 mT). The sample was then rotated in the constant field, and Fresnel mode LTEM images were acquired at each field angle.

### Electron holography

Off-axis transmission electron holography (EH) is an interferometric technique based on the Aharanov-Bohm effect, where the phase shift of an initially coherent electron beam is recorded when interacting with the electromagnetic fields within and around the sample. For off-axis EH experiments in the TEM, the beam is split into two parts, where one part of the beam passes through vacuum (i.e. the reference beam whose phase remains unchanged), while the other part interacts with the sample inducing a phase change. The two parts on the beam are then deflected using a Möllenstedt biprism to overlap the reference and sample beam to create an interference pattern, i.e. the hologram, which contains both the intensity information that is created in a conventional TEM image, and local phase variations. Using Fourier analysis, one can extract the local phase shifts from the hologram and thus map the in-plane magnetic induction within the sample.

EH was performed using the dedicated Hitachi HF-3300 (I2TEM) microscope operated at 300 kV, using a double biprism setup. The holograms were acquired using a homemade corrective software to correct for both a drift in interference fringes and of the sample^59^ with an exposure time of 60 s per hologram and an inter-fringe distance of 1.4 nm. The resulting resolution of the treated magnetic phase images extracted by homemade software is 3.7 nm.

### Image analysis

To create the datasets in Fig. 1, image segmentation was required to identify the individual switching fields for each NI. For this, the components of the lattice were segregated based on intensity and size to form a mask for each NI away from the image boundaries. This was performed using image segmentation tools, available in MATLAB^®^, by a morphological reconstruction method^60^. Throughout the LTEM field-sequence, the intensity on either side of each NI major axis was sampled to establish the field where the magnetization was reversed by an inversion of intensity. The DS lengths were measured manually for the fields shown.

### Micromagnetic modeling

OOMMF^46^ is a finite difference method solver that calculates the total energy of regularly distributed discretized cells, by the LLG equation, over the object domain by considering the summed influence of different micromagnetic energies on objects with predetermined magnetization. The magnetization within each cell is updated by a conjugate gradient energy evolver, which calculates the local energy minima directly. The energy-evolver is ideal for computationally larger structures, such as ASI lattices, as it allows the problem to reach convergence far quicker than the time-evolvers with the same result.

The component sizes of the modeled structures were conserved between simulations and experiments. The structure modeled in Fig. 3 was 5216 nm × 5216 nm in size and replicated the full structure depicted in Fig. 2; whereas the array modeled in Figs. 4, 5 was reduced to 12.042 µm × 12.042 µm to reduce simulation times and memory. The magnetic parameters of Py used for the micromagnetic modelling were taken from literature, *M*_s_ = 800 × 10^3^ A/m and *J*_ex_ = 13 × 10^−12^ J/m, and the regularized mesh was comprised of 3 × 3 nm cells to best resolve the NI features.

## Supplementary information

Supplementary Information

Description of Additional Supplementary files

Supplementary Movie 1

Supplementary Movie 2

Supplementary Movie 3

Supplementary Movie 4

Supplementary Movie 5

Supplementary Movie 6

Supplementary Movie 7

## Data Availability

The LTEM data from Fig. 1 and Supplementary Figs. 1–3 have been provided in Supplementary Movies 1–6. Additional data generated in this study has been deposited in the Zenodo repository (6563587) or are available from the corresponding author upon reasonable request.

## Code availability

The authors declare that all codes used for the analysis presented in this study are available from the corresponding author upon reasonable request.

## References

[1] Wang RF (2006). Artificial ‘spin ice’ in a geometrically frustrated lattice of nanoscale ferromagnetic islands. Nature.

[2] Tanaka M, Saitoh E, Miyajima H, Yamaoka T, Iye Y (2006). Magnetic interactions in a ferromagnetic honeycomb nanoscale network. Phys. Rev. B - Condens. Matter Mater. Phys..

[3] Skjærvø SH, Marrows CH, Stamps RL, Heyderman LJ (2020). Advances in artificial spin ice. Nat. Rev. Phys..

[4] Bramwell ST (2001). Spin ice state in frustrated magnetic pyrochlore. Mater. Sci. (80-.)..

[5] Bramwell, S. T. & Harris, M. J. The history of spin ice. *J. Phys. Condens. Matter* **32**, (2020). 10.1088/1361-648X/ab8423 32554893

[6] Thonig, D., Reißaus, S., Mertig, I. & Henk, J. Thermal string excitations in artificial spin-ice square dipolar arrays. *J. Phys. Condens. Matter* **26**, (2014). 10.1088/0953-8984/26/26/266006 24912993

[7] Anghinolfi L (2015). Thermodynamic phase transitions in a frustrated magnetic metamaterial. Nat. Commun..

[8] Perrin Y, Canals B, Rougemaille N (2016). Extensive degeneracy, Coulomb phase and magnetic monopoles in artificial square ice. Nature.

[9] Macauley GM (2020). Tuning magnetic order with geometry: thermalization and defects in two-dimensional artificial spin ices. Phys. Rev. B.

[10] Schánilec V (2020). Bypassing dynamical freezing in artificial kagome ice. Phys. Rev. Lett..

[11] Gliga S (2017). Emergent dynamic chirality in a thermally driven artificial spin ratchet. Nat. Mater..

[12] Lehmann J, Donnelly C, Derlet PM, Heyderman LJ, Fiebig M (2019). Poling of an artificial magneto-toroidal crystal. Nat. Nanotechnol..

[13] Pohlit M (2020). Collective magnetic dynamics in artificial spin ice probed by ac susceptibility. Phys. Rev. B.

[14] Puttock R (2020). Modal frustration and periodicity breaking in artificial spin ice. Small.

[15] Bhat VS, Heimbach F, Stasinopoulos I, Grundler D (2016). Magnetization dynamics of topological defects and the spin solid in a kagome artificial spin ice. Phys. Rev. B.

[16] Haldar A, Kumar D, Adeyeye AO (2016). A reconfigurable waveguide for energy-efficient transmission and local manipulation of information in a nanomagnetic device. Nat. Nanotechnol..

[17] Ghosh A (2020). Emergent dynamics of artificial spin-ice lattice based on an ultrathin ferromagnet. Nano Lett..

[18] Gliga S, Iacocca E, Heinonen OG (2020). Dynamics of reconfigurable artificial spin ice: toward magnonic functional materials. APL Mater..

[19] Kaffash MT, Lendinez S, Jungfleisch MB (2021). Nanomagnonics with artificial spin ice. Phys. Lett. A.

[20] Heyderman LJ, Stamps RL (2013). Artificial ferroic systems: novel functionality from structure, interactions and dynamics. J. Phys. Condens. Matter.

[21] Nisoli C, Moessner R, Schiffer P (2013). Colloquium: artificial spin ice: designing and imaging magnetic frustration. Rev. Mod. Phys..

[22] Vedmedenko EY (2016). Dynamics of bound monopoles in artificial spin ice: how to store energy in dirac strings. Phys. Rev. Lett..

[23] Arava, H. et al. Computational logic with square rings of nanomagnets. *Nanotechnology* **29**, (2018). 10.1088/1361-6528/aabbc3 29620015

[24] Jensen, J. H., Folven, E. & Tufte, G. Computation in artificial spin ice. in *The 2018 Conference on Artificial Life* 15–22 (MIT Press, 2018). 10.1162/isal_a_00011.

[25] Arava H (2019). Engineering relaxation pathways in building blocks of artificial spin ice for computation. Phys. Rev. Appl..

[26] Sanz-Hernández D (2021). Tunable stochasticity in an artificial spin network. Adv. Mater..

[27] Castelnovo C, Moessner R, Sondhi SL (2008). Magnetic monopoles in spin ice. Nature.

[28] Ladak S (2010). Direct observation of magnetic monopole defects in an artificial spin-ice system. Nat. Phys..

[29] Mengotti E (2011). Real-space observation of emergent magnetic monopoles and associated Dirac strings in artificial kagome spin ice. Nat. Phys..

[30] Mellado P, Petrova O, Shen Y, Tchernyshyov O (2010). Dynamics of magnetic charges in artificial spin ice. Phys. Rev. Lett..

[31] Ladak S, Read D, Tyliszczak T, Branford WR, Cohen LF (2011). Monopole defects and magnetic Coulomb blockade. N. J. Phys..

[32] Ladak, S. et al. Disorder-independent control of magnetic monopole defect population in artificial spin-ice honeycombs. *New J. Phys*. **14**, (2012).

[33] Zeissler, K. Magnetic and electrical transport properties of artificial spin ice. (Imperial College London, 2013).

[34] Srivastava A (2015). Optically active quantum dots in monolayer WSe2. Nat. Nanotechnol..

[35] dos Santos RM, de Aguiar AL, Ribeiro Junior LA, da Rocha Martins J (2019). Electronic and structural properties of vacancy endowed BCN heterostructures. Chem. Phys. Lett..

[36] Drisko J, Marsh T, Cumings J (2017). Topological frustration of artificial spin ice. Nat. Commun..

[37] Lau GC (2006). Zero-point entropy in stuffed spin-ice. Nat. Phys..

[38] Revell HM (2013). Evidence of impurity and boundary effects on magnetic monopole dynamics in spin ice. Nat. Phys..

[39] Sala G (2014). Vacancy defects and monopole dynamics in oxygen-deficient pyrochlores. Nat. Mater..

[40] Bowman, D. F. et al. Role of defects in determining the magnetic ground state of ytterbium titanate. *Nat. Commun*. **10**, (2019). 10.1038/s41467-019-08598-z PMC6367421 30733436

[41] Möller G, Moessner R (2006). Artificial Square Ice and Related Dipolar Nanoarrays. Phys. Rev. Lett..

[42] Shigeto K, Shinjo T, Ono T (1999). Injection of a magnetic domain wall into a submicron magnetic wire. Appl. Phys. Lett..

[43] Thomas L (2005). Observation of injection and pinning of domain walls in magnetic nanowires using photoemission electron microscopy. Appl. Phys. Lett..

[44] McGrouther D, McVitie S, Chapman JN, Gentils A (2007). Controlled domain wall injection into ferromagnetic nanowires from an optimized pad geometry. Appl. Phys. Lett..

[45] Lichte, H. & Lehmann, M. Electron holography - Basics and applications. *Reports Prog. Phys*. **71**, (2008).

[46] Porter, D. G. & Donahue, M. J. *OOMMF User’s Guide, Version 1.0*. http://math.nist.gov/oommf (1999).

[47] Miltat, J. E. & Donahue, M. J. Numerical Micromagnetics: Finite Difference Methods. *Handb. Magn. Adv. Magn. Mater*. 10.1002/9780470022184.hmm202. (2007).

[48] Gliga S, Kákay A, Heyderman LJ, Hertel R, Heinonen OG (2015). Broken vertex symmetry and finite zero-point entropy in the artificial square ice ground state. Phys. Rev. B.

[49] Koltsov DK, Cowburn RP, Welland ME (2000). Micromagnetics of ferromagnetic equilateral triangular prisms. J. Appl. Phys..

[50] Thevenard L, Zeng HT, Petit D, Cowburn RP (2009). Six-fold configurational anisotropy and magnetic reversal in nanoscale Permalloy triangles. J. Appl. Phys..

[51] Morgan JP, Stein A, Langridge S, Marrows CH (2011). Thermal ground-state ordering and elementary excitations in artificial magnetic square ice. Nat. Phys..

[52] Jungfleisch MB (2017). High-frequency dynamics modulated by collective magnetization reversal in artificial spin ice. Phys. Rev. Appl..

[53] Morley SA (2017). Representation of compounds for machine-learning prediction of physical properties. Phys. Rev. B.

[54] Pancaldi M, Leo N, Vavassori P (2019). Selective and fast plasmon-assisted photo-heating of nanomagnets. Nanoscale.

[55] Morley SA (2019). Thermally and field-driven mobility of emergent magnetic charges in square artificial spin ice. Sci. Rep..

[56] Gartside JC (2018). Realization of ground state in artificial kagome spin ice via topological defect-driven magnetic writing. Nat. Nanotechnol..

[57] Wang Y-L (2016). Rewritable artificial magnetic charge ice. Sci. (80-.).

[58] Arava H (2020). Control of emergent magnetic monopole currents in artificial spin ice. Phys. Rev. B.

[59] Gatel C, Dupuy J, Houdellier F, Hÿtch MJ (2018). Unlimited acquisition time in electron holography by automated feedback control of transmission electron microscope. Appl. Phys. Lett..

[60] Vincent L (1993). Morphological grayscale reconstruction in image analysis: applications and efficient algorithms. IEEE Trans. Image Process.

