# Supplementary material for: Defect-induced monopole injection and manipulation in artificial spin ice
Source: Nat Commun. 2022 Jun 25;13:3641. doi: 10.1038/s41467-022-31309-0 (PMC9233697; doi:10.1038/s41467-022-31309-0)
Supplement: Supplementary file 1 — Supplementary Information [file 41467_2022_31309_MOESM1_ESM.pdf]

Supplementary Information: Defect-induced monopole injection and manipulation in artificial spin ice

R. Puttock *et al.*

Corresponding Author: robb.puttock@npl.co.uk

Supplementary Note 1: ASI with Solid defect:

In Figure 1b, we show a masked differential LTEM image to represent macrospin flips in the lattice with solid defect under field reversal. Figure SUPPLEMENTARY FIGURE1 shows the raw differential LTEM image (i.e. sequentially subtracted between images rather than masked) for a select iterations of the field sequence shown in the manuscript (a) and an additional differential LTEM image sequence along the opposite field direction (b). For the full dataset, please see Supplementary Movies 1 and 2. The field direction was applied approximately 37° from the image horizontal.

One item of note here is the non-symmetric field reversal (i.e. horizontal direction in (a), and diagonal in (b)). This effect is not a result of the defect presence as it was also replicated with the vacant defect lattice (see Supplementary Note 2). Instead, we suspect this was an effect from good alignment of the field angle with the armchair axis, which does introduce some stochasticity in the propagation direction.

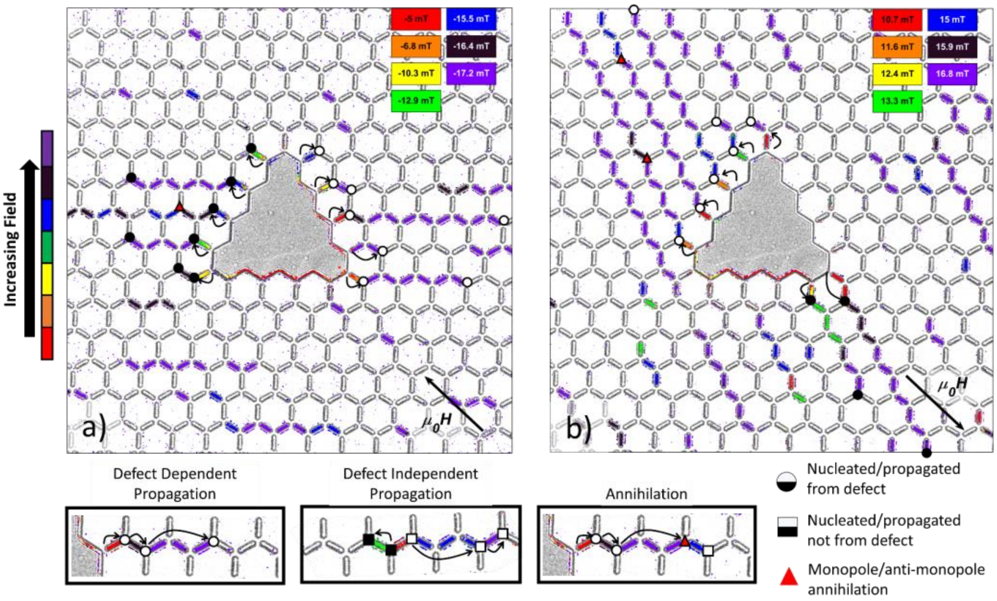

Supplementary Fig. 1. (a-b) Armchair direction: *Differential LTEM images of Defect-ASI lattice under field reversal (negative and positive directions, respectively) along armchair direction; colors indicate the field applied when nanoislands undergo magnetization reversal. Symbols are described by the key below the image.*

Supplementary figure 2 (a-b) shows the field reversal of the lattice with a solid defect as the field is applied down the zig-zag axis. The sample was rotated 30° and the field is still applied 37° from the image horizontal. Here, the same effects in islands near the defect reverse at lower fields, but more island switches in the bulk of the lattice is also observed from the favorable field alignment along the zig-zag axis. Videos of the full field reversal are presented in supplementary videos Supplementary Movies 3 and 4, respectively.

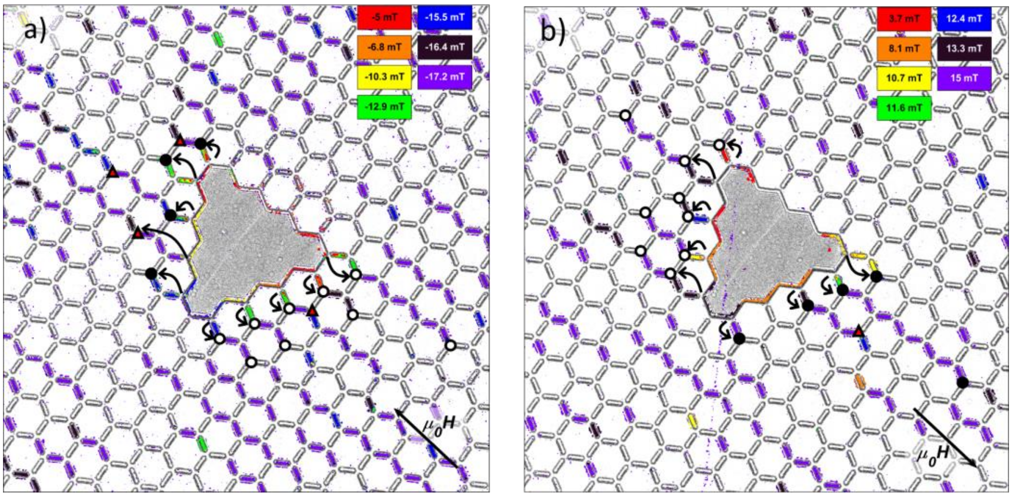

Supplementary Fig. 2. (a-b) zigzag direction: *Differential LTEM images of Defect-ASI lattice under field reversal (negative and positive directions, respectively) along zigzag direction; colors indicate the field applied when nanoislands undergo magnetization reversal.*

Supplementary Note 2: ASI with vacant defect.

In Figure 1e, we show a masked differential LTEM image to represent macrospin flips in the lattice with vacant defect under field reversal. Supplementary Figure 3 shows the raw differential LTEM image for select iterations of the field sequence shown in the manuscript (a) and an additional differential LTEM image sequence along the opposite field direction (b). For the full dataset, please see supplementary videos SV5 and SV6. The field direction was applied approximately 37° from the image horizontal. The field sequence and the image analysis has been kept consistent between Supplementary Figure 1 and 3 for direct comparison.

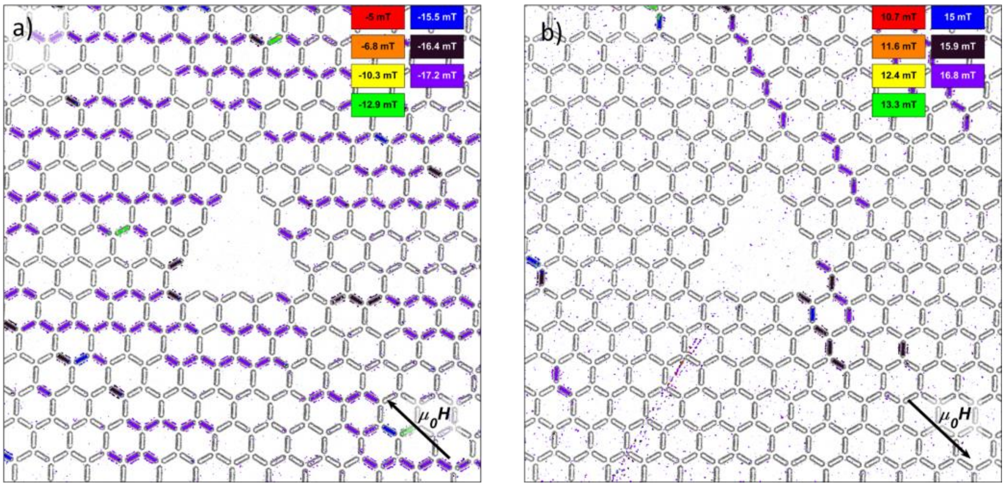

Supplementary Fig. 3. *(a-b) Differential LTEM images of vacant ASI lattice under field reversal (negative and positive directions, respectively) along the armchair direction; colors indicate the field applied when nanoislands undergo magnetization reversal.*

Supplementary Note 3. Micromagnetic modelling Defect island distances

This section addresses the effects of the defect's shape anisotropy on the interaction with nearest neighbor NIs. Figure 2e of the main manuscript shows how the demagnetization energy (E_d) of a single NI and defect system changes with defect-island separation. Supplementary Figure 4(a) plots E_d as a function of island distance from the defect for three islands in different positions around the saturated

defect. The NIs are magnetized antiparallel to the field direction ($\mu_0 H_{||}$ = 20 mT) and the saturated defect. The island at the focused end of the defect (relative to the field direction) experiences a notably greater force from the saturated defect than those at the "base" of the triangular defect. This demonstrates the anisotropy of MP propagation between the tapered end and the base and explains why MPs at the base appear less mobile than those at the tip in this field orientation.

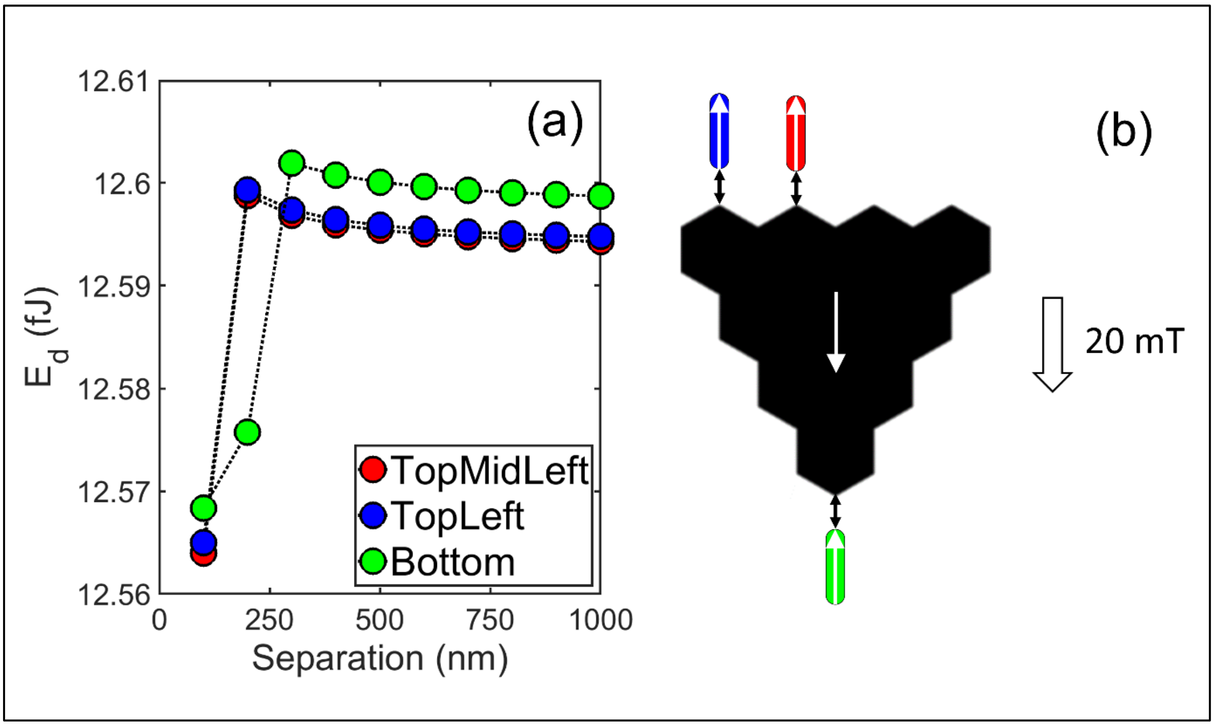

Supplementary Fig. *4. (a) Modeled demagnetization energy (E_d) of the saturated defect and single islands at positions around the defect indicated by the schematic in (b). The NIs are initially magnetized anti-parallel (to the applied field ($\mu_0 H_{||}$ = 20 mT) and plotted as a function of defect-island separation.*

Supplementary Note 4. Micromagnetic modelling of the small Defect-ASI lattice

In Figure 3 in the manuscript, we show the results from micromagnetic modelling of a smaller lattice with defect in the center under field reversal. We focused on the top left corner to better visualize the coupling across the defect-lattice interface. Supplementary Figure 5 displays the magnetization maps of the full structure at the same snapshots that were displayed in Figure 3 of the main manuscript. Here, the color represents the angle of the magnetization vector from the x-axis (see color wheel, inset). Supplementary Movie 7 presents a gif of the full modeled field reversal starting from the state depicted in (a) and ending at the state depicted in (e) at 5 mT iteration steps.

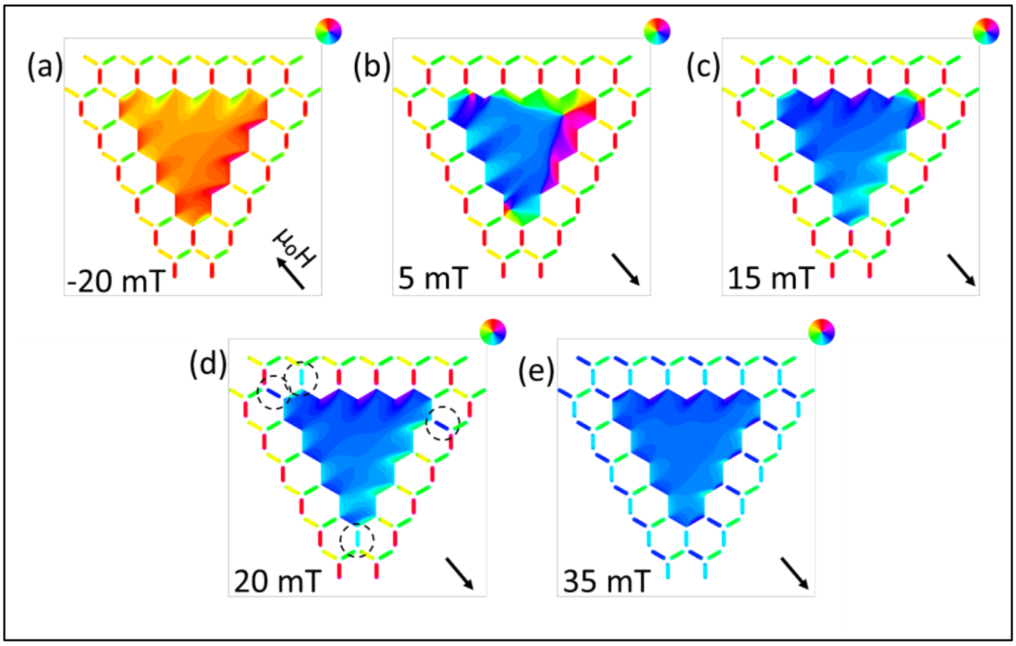

Supplementary Fig. 5. (a-e) *Modeled magnetization configuration (color represents angle of the magnetic vector with respect to the x-axis) of the full small defect-ASI lattice as it undergoes magnetization reversal along the indicated sweep direction. Switched islands in (d) are circled.*

Supplementary Note 5. Determining the shape anisotropy of the DASI lattice

To investigate the anisotropic properties of the Defect-ASI lattice, we have performed micromagnetic modelling analysis of the remanence magnetisation (M_r) as a function of the applied field angle (θ). Three systems were investigated:

1. ASI lattice only (with vacant defect)
2. Defect only
3. Defect-ASI lattice

For optimised speed of the simulations the lattices were based on the small DASI lattice were used, meaning that there is a greater influence of lattice boundary effects. The three systems are depicted in Supplementary Figure 6(a-c), respectively. In the first step each system is saturated in $\mu_0 H_{\parallel}$ = 2 T field along angle θ, measured from the *y*-axis (in 2° iteration steps). To better match the experimental method, we also included a fixed out-of-plane field of $\mu_0 H_{\perp}$ = 0.488 T. In the second step, the total field is removed, and the system evolves towards remanence.

The M_r polar plots were presented in Fig. 4b of the manuscript. Supplementary Figure 6(d) presents the magnetostatic energy (E_d) of the remanence state for the three systems, and the combined E_d by totalling the individual defect and lattice components for comparison. The defect has clear higher E_d remanence configurations when the field is aligned approximately parallel to its triangular edges. The lattice E_d from the remanence configuration lattice is practically flat by comparison as the shape anisotropy results in a settled configuration of degenerate energy configurations as is expected for

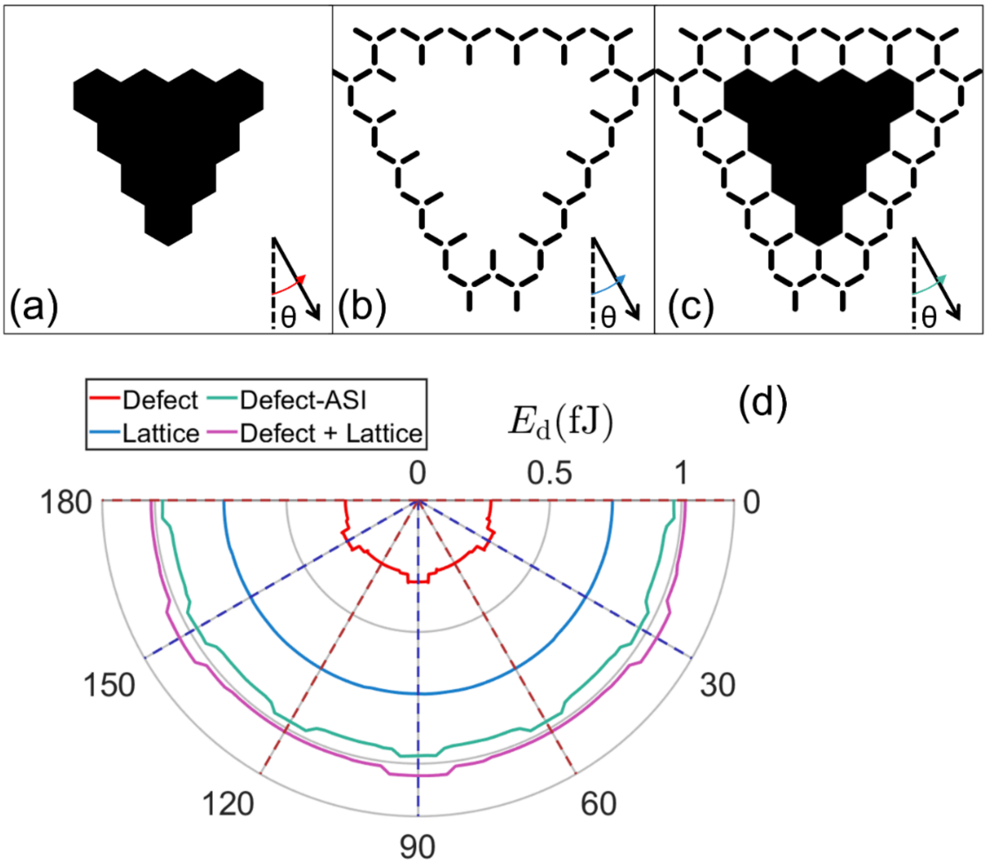

Supplementary Fig. 6. *(a-c) Masks of the three structures investigated under the micromagnetic modelling (defect, Kagome lattice without defect, and Kagome lattice with defect, respectively. (d) Polar plot of the magnetostatic energy at zero-field remanence after saturating along field angle, θ ($\mu_0 H_{\parallel}$ = 2 T), for the three structures in (a-c) and the combination of (a) + (b) (for the defect + lattice plot) (red, blue, green, and purple, respectively).*

ASI. The defect-ASI lattice has distinct E_d peaks that are not congruent with the individual components of Supplementary Figure 6a and b when the field is aligned along the defect symmetry axis and lattice armchair axis. This is highlighted by comparing with the combined plot (purple) where this profile is not present. It represents an extra metastable configuration where the buckle domain state is not formed from the defect-ASI coupling. Instead, the ASI lattice promotes a completely different magnetic configuration in the defect. Supplementary Figure 7 present the magnetic magnetisation maps for the three systems at the key field angles at remanence to demonstrate this fact.

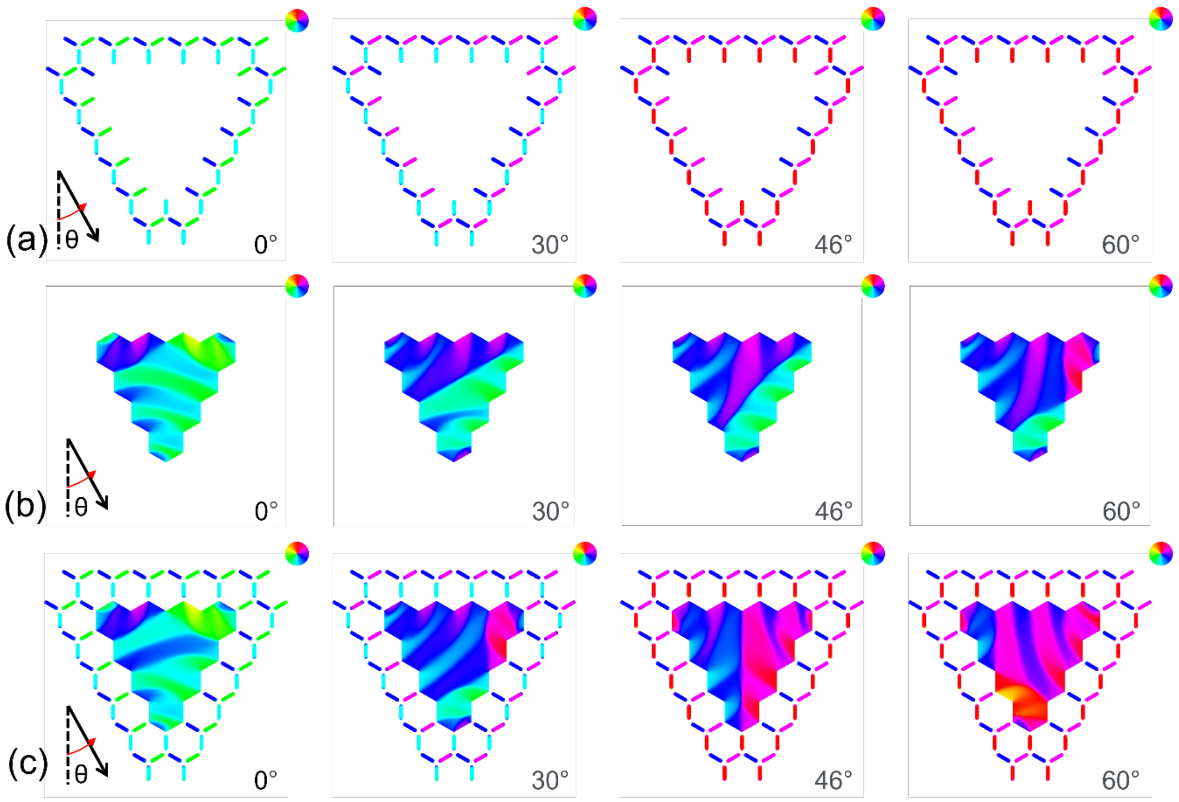

***Supplementary Fig. 7**. (a-c) Modelled magnetization maps of the three structures depicted in Fig. SF6(a-c) at zero-field remanence after saturation along field angles* θ = 0°, 30°, 46°, *and* 60°.

Supplementary Note 6. Angular propagation in defect-ASI lattice.

In Figure 4 of the main manuscript, we show through micromagnetic modelling how small variations in the field angle at a magnitude below the switching field of the lattice can result in controlled propagation of the defect-bound monopoles. Here, we show a few further examples of different field histories and the effects this can have on the monopole propagation.

Supplementary Figure 8 (a-b) presents the magnetization maps of the defect-ASI lattice when the field, $\mu_0H_{\|}$, is applied along the zigzag and armchair axes, respectively. The in-plane field magnitude was $\mu_0H_{\|}$ = 15 mT, 20 mT and 25 mT (i-iii, respectively) and the starting state of the lattice in all data was at zero-remanence after initially saturation to the left of the image. The quantity of nucleated defect-bound monopoles Supplementary Fig. 8(a-b) is maximized with an applied field parallel to the defect edge. When the field is perfectly aligned with the zigzag axis (a), the defect bound monopoles are pinned on +/-*q* sites in the lattice. This is because the neighboring sites would be +/- 3q sites if it was where the monopole resided, making them slightly less favorable. This is also why black monopoles (right of the defect) settle two sites away from the defect, whereas white monopoles are pinned at one site away from the defect. When the field is aligned perfectly with the armchair axis (b) the symmetry creates an effectively deep saddle point that makes monopole propagation less favorable, even though the black monopoles (right of the defect) are fixed on +3q sites. In real systems there is rarely perfect symmetry at the lattice junctions from extrinsic factors or material variations, which would enable propagation somewhat stochastically.

Supplementary Fig. 8(c) demonstrates the importance of misalignment from the armchair and zigzag axes when controlling the defect-bound monopoles. The initial configuration of the lattice is that depicted in Supplementary Fig. 8b(iii), when the field, $\mu_0H_{\|}$ = 25 mT, is applied along the armchair axis

at β = 30°. The field is rotated in 1° iterations towards the zigzag axis at β = 0°. Supplementary Fig. 8(c) depicts the differential map where switched islands are shaded according to the color-scheme provided (right). When the field is applied off-axis, the monopoles at the top-right of the defect propagate to the edge of the lattice, where they terminate. The defect's influence on the defect-bound monopoles decays as you look down the defect as the symmetry is broken with respect to the field reorientation and the defect field strength is maximised near at the corners on the defect edge that is parallel to the zigzag axis. The asymmetry in propagation between the left and right sides of the defect is clearly seen in Supplementary Fig. 8(c).

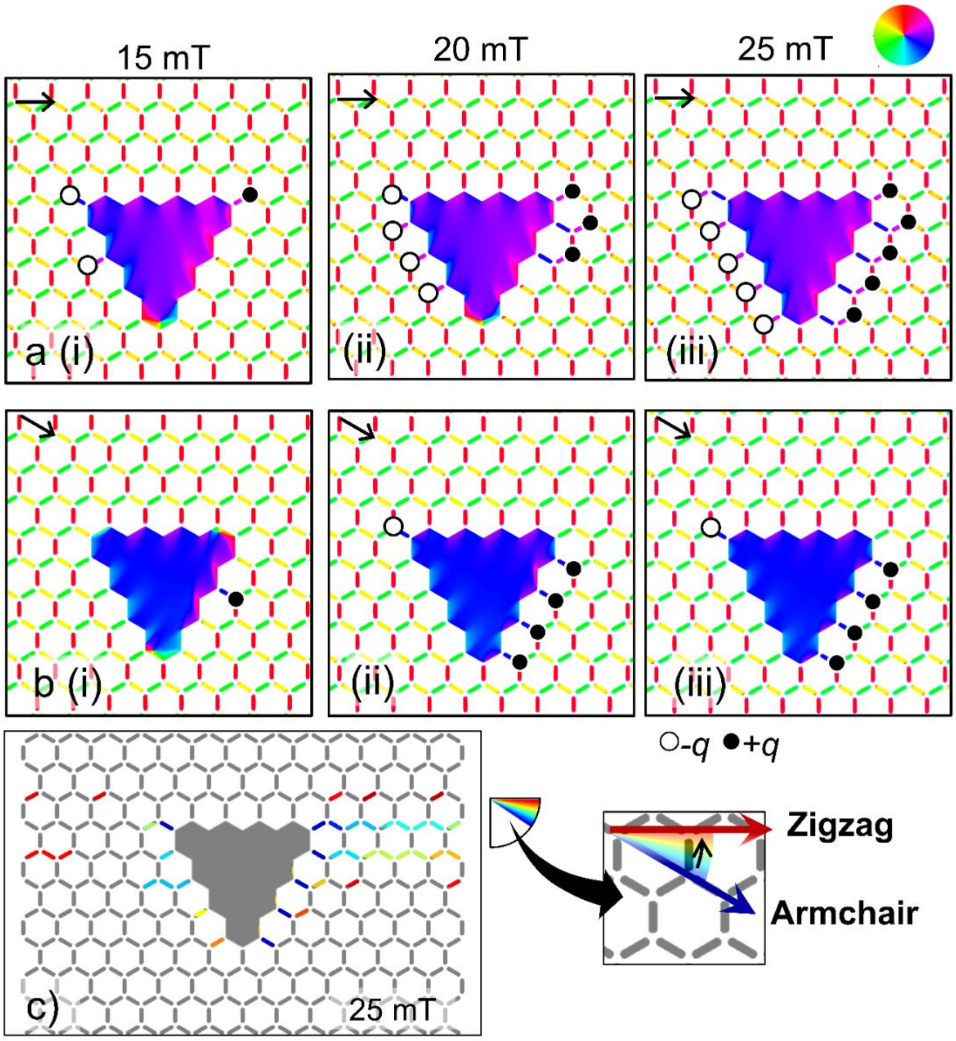

Supplementary Fig. 8.(a-b) *Magnetization maps of defect-ASI when field is applied down the zigzag (0°) and armchair (30°) axes, respectively, for in-plane field magnitude μ0H|| = 15 mT, 20 mT and 25 mT (i-iii, respectively). (c) Differential map of modelled switching events in the defect-ASI lattice as the field is rotated from the initial armchair direction to the zigzag axis under field* $\mu_0 H_{||}$ *= 25 mT. Colored islands represent the switching angle according to the colorscale (right).*

Supplementary Figure 9 shows modelled differential images of propagation under different field histories (keeping the field magnitude constant), starting from the initial state depicted in Figure 4(b) in the main manuscript (represented by red shading in a-c). Supplementary Fig. 9(a) displays the same dataset as is shown in Figure 4(e) in the main manuscript, where only the monopoles to the top-left of the defect propagate under the teetering field.

Supplementary Figure 9(b) instead tilts the field towards the image vertical (θ=60°) in the 2nd step, where there is more favorable alignment with the y-oriented nanoislands. As a result, there are two nucleation events at the top of the defect and one propagation event to the right of the defect (shaded purple). Under more field-steps, we see more of the defect-bound monopole charges propagating (blue and cyan). Supplementary Figure 9(c) shows one more example where the field is rotated further towards the image horizontal (θ=41°) than in Supplementary Figure 9(a) in the 2nd step, resulting in extra propagation events in defect-bound monopoles to the right of the defect.

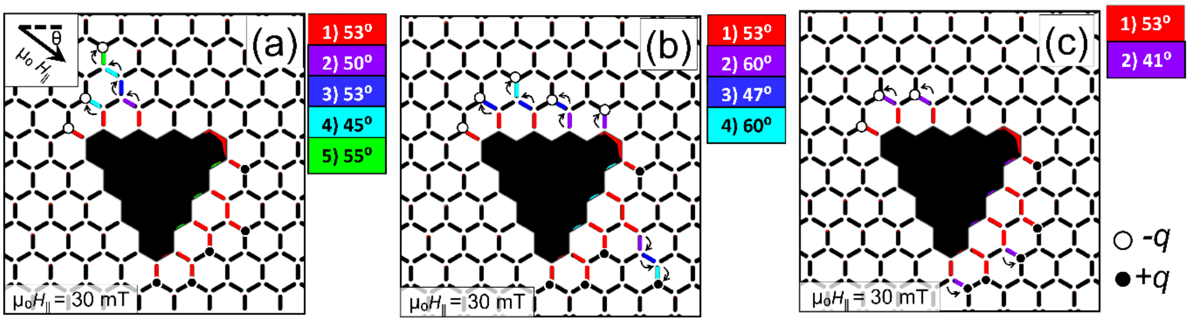

Supplementary Fig. 9. (a) *Differential map of modelled switching events in the defect-ASI lattice as the field angle* (θ) *is modified within 45° - 55° at field magnitude,* μ_0H = 30 mT. *(b-c) Differential map of modelled switching events where the field is rotated towards and away from the image vertical, respectively, in the 2nd step to encourage more propagation events. Color shading indicates a change in the magnetization direction between images when the change in each discretized cell >* 100°.

Supplementary Figure 10(a-b) displays the differential image displayed in Supplementary Figure 9(a) (and Figure 4(d) in the main manuscript) with an additional field step applied along the lattice horizontal in the positive and negative directions, respectively. The additional step in both (a) and (b) is colored yellow.

Field application along θ = 0° (Supplementary Fig. 10(a)) favors more nucleation and propagation events of defect-bound monopoles along this field direction as it coincides with one of the zigzag axes and acts in the opposite direction to the initial magnetization state. Some monopoles that have formed between steps #1-5 are unchanged in this step because of the inhomogeneous charge topology of the Kagome lattice[1,2]. This means that when a monopole sits on a ±q site it is slightly less favorable to propagate onto a ±3q site. Whereas those that already sit on a ±3q vertex site will more readily propagate onto a ±q site (see schematic). This additional energy barrier is overcome by more favorable alignment of the field along the desired pathway, as shown in Supplementary Figure 9.

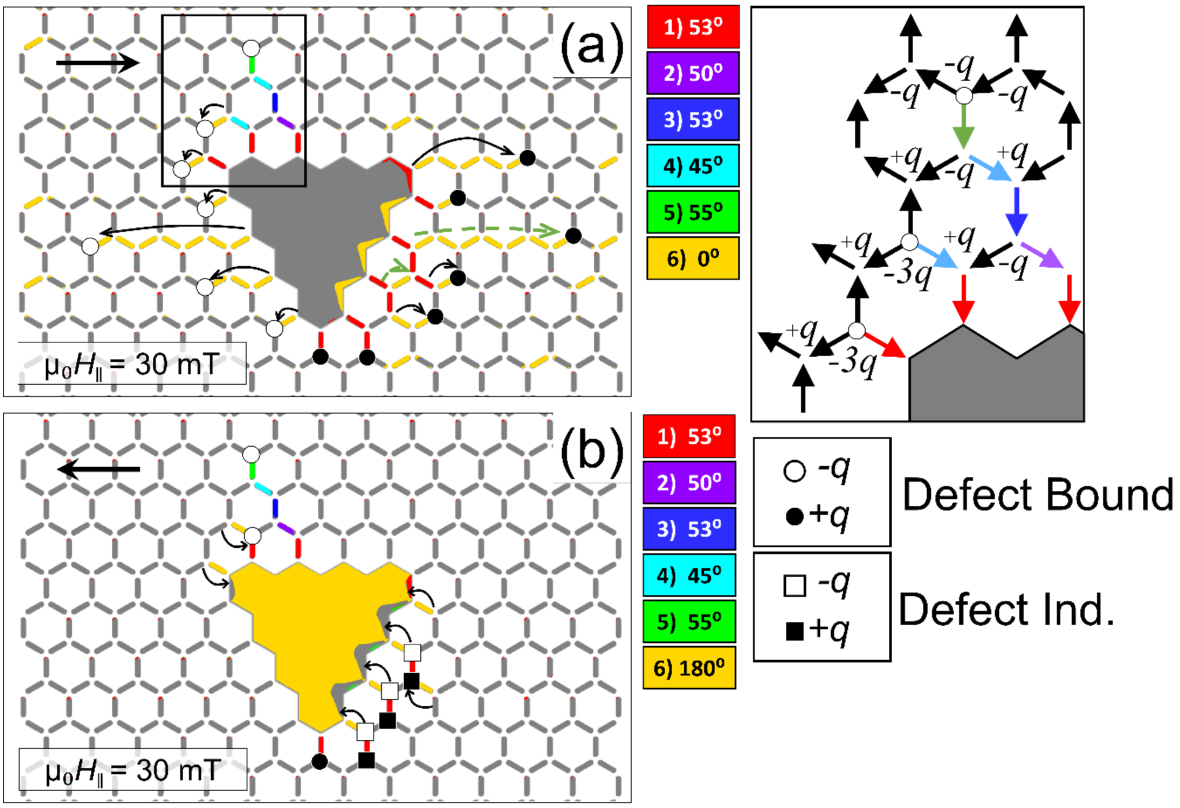

Supplementary Fig. 10. *Differential maps of modeled switching events in the defect-ASI lattice with the field angle $\vartheta = 0^o$ and $\vartheta = 180^o$ (a and b, respectively) in the final step with field magnitude $\mu_0 H = 30$ mT. Right: vertex charge and macrospin schematic at step 5 for the initial state of the region highlighted in (a), explaining why it is favorable for some monopole vertices to switch. Color shading indicates a change in the magnetization direction between images when the change in each discretized cell > 100°. Topologically allowed monopole "branching" in (a) is indicated by green-dashed arrows[3].*

In Supplementary Figure 10(b) the field was instead applied along the $\theta = 180^o$ direction. Although this is still along the zigzag axis, many of the islands in the wider lattice are already aligned with the field. It results in the switched nanoislands on this axis to realign with the field direction. Nanoislands aligned orthogonal to the field are unperturbed at this field strength. This has the effect of either shortening the length of the Dirac string (e.g. top left of defect); forming defect-independent monopoles (e.g. bottom right of defect); or "reabsorbing" the monopole (e.g. top right of defect).

Supplementary Figure 11 shows the magnetization maps of the DASI lattice as an increasing field is applied along the *x*-axis ($\mu_0 H$ = 30-35 mT) starting from the state depicted in Supplementary Figure 9(a). For simplicity, only the monopoles that are bound to the defect are drawn onto the magnetization maps (white/black circles). Upon increasing the applied field, both the monopoles that were formed originally and those that have newly formed with the change in field direction, propagate to the lattice edges (a-d). This includes annihilations with other independent MP-AMP pairs. In Supplementary Figure 11(e) we see the lattice has almost fully saturated except for a few stubborn islands around the original DSs, which have been marked with boxes. This is because of the coincidental formation of chiral flux-closing configurations as depicted in the diagrams of the same regions in Supplementary Figure 12a(i) and b(i) (top and bottom boxes of Supplementary Figure 10e, respectively). This requires a bit more energy to overcome, as depicted in Supplementary Figure 11(f) and 12a(ii) and b(ii) when $\mu_0 H$ = 35 mT.

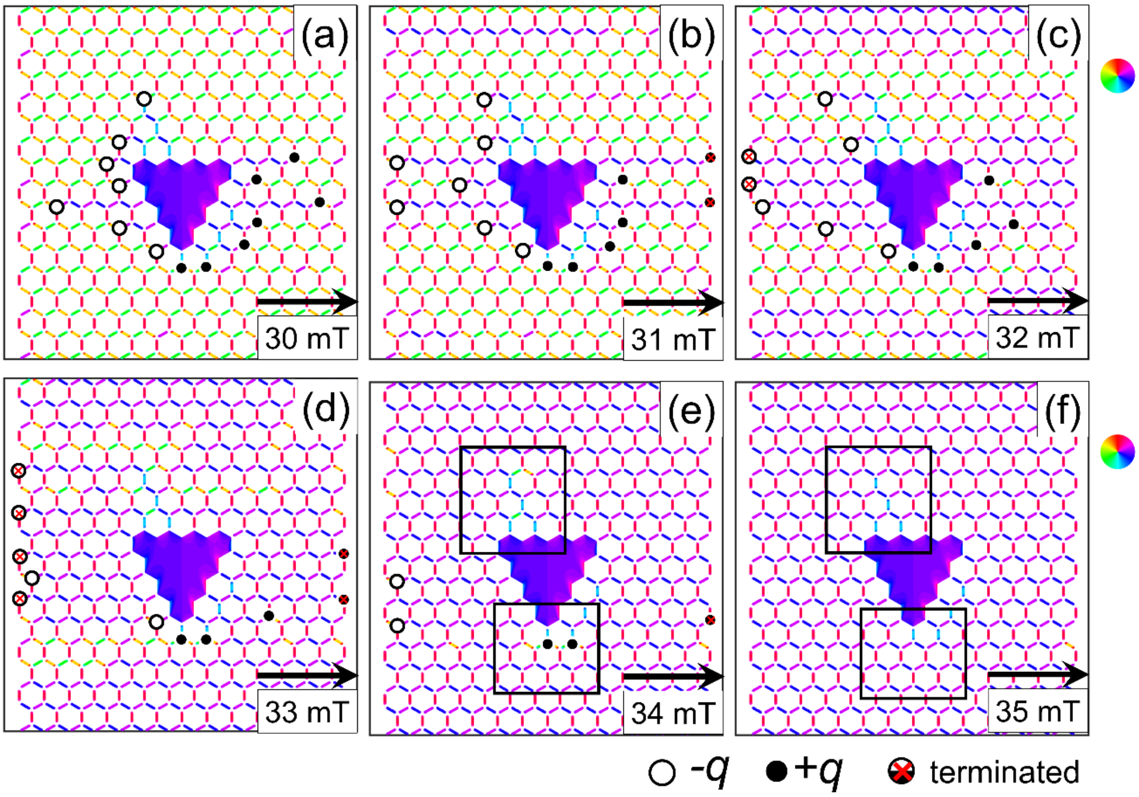

Supplementary Fig. 11. *Magnetization maps of DASI starting from the state depicted in Fig. SF5(a) under increasing field (30-35 mT) applied along the x-axis. Defect-bound Monopole charges are indicated according to the key provided. Boxes in (e-f): areas where islands perpendicular to the field direction are conserved after saturation along the zig-zag axis and correlate to schematics in SF12 a-b (i-ii), respectively.*

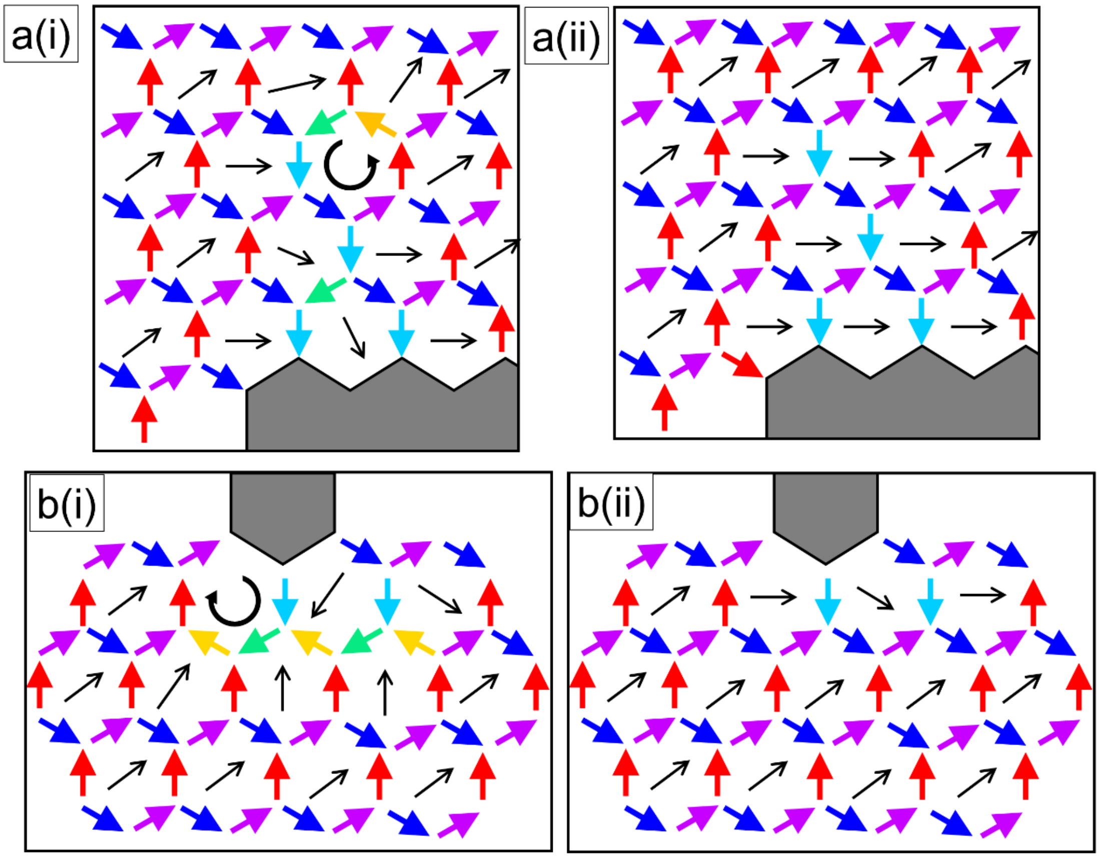

Supplementary Fig. 12. *(a-b) Schematics of the magnetic configurations depicted in SF 7e-f, respectively. Colored arrows indicate the island magnetization direction, and the black arrows indicate the net magnetization of each hexagonal unit cell. The formation of spirals/vortexes create very stable configurations that require more field to overcome.*

Supplementary Note 7. Repeated field propagation in LTEM

Supplementary Figure 13 presents repeat measurements to compliment the field teetering measurements displayed in Figure 5. Here, we show that the monopoles that propagate from the defect are repeatable, with regard to positioning and distance, before MP-AMP pairs are formed in the ASI lattice. In all cases, the final steps displayed (red islands) are the last possible before cascade style switching events across the lattice occurred.

Supplementary Figure 14 presents the switching events in the sample when it is continuously rotated under a constant field from θ = 29-59°. The lattice is initially saturated towards its top-left corner of the image, followed by the subsequent application of $\mu_0 H_{\|} \approx 21$ mT at θ = 29° from the lattice horizontal, where the initial switched NIs are represented by blue. Navy-blue NIs away from the defect are states that were frozen into the lattice from insufficient initialization by saturation but are included for completion. The long chain is the result of numerous annihilation events as the monopoles. As one end is fixed, the annihilation event results in a defect-bound monopole very far from the defect location. This indicates that, regardless of annihilation events in the lattice, there will be a Dirac string of inverted dipoles linking the defect to the final position of the monopole.

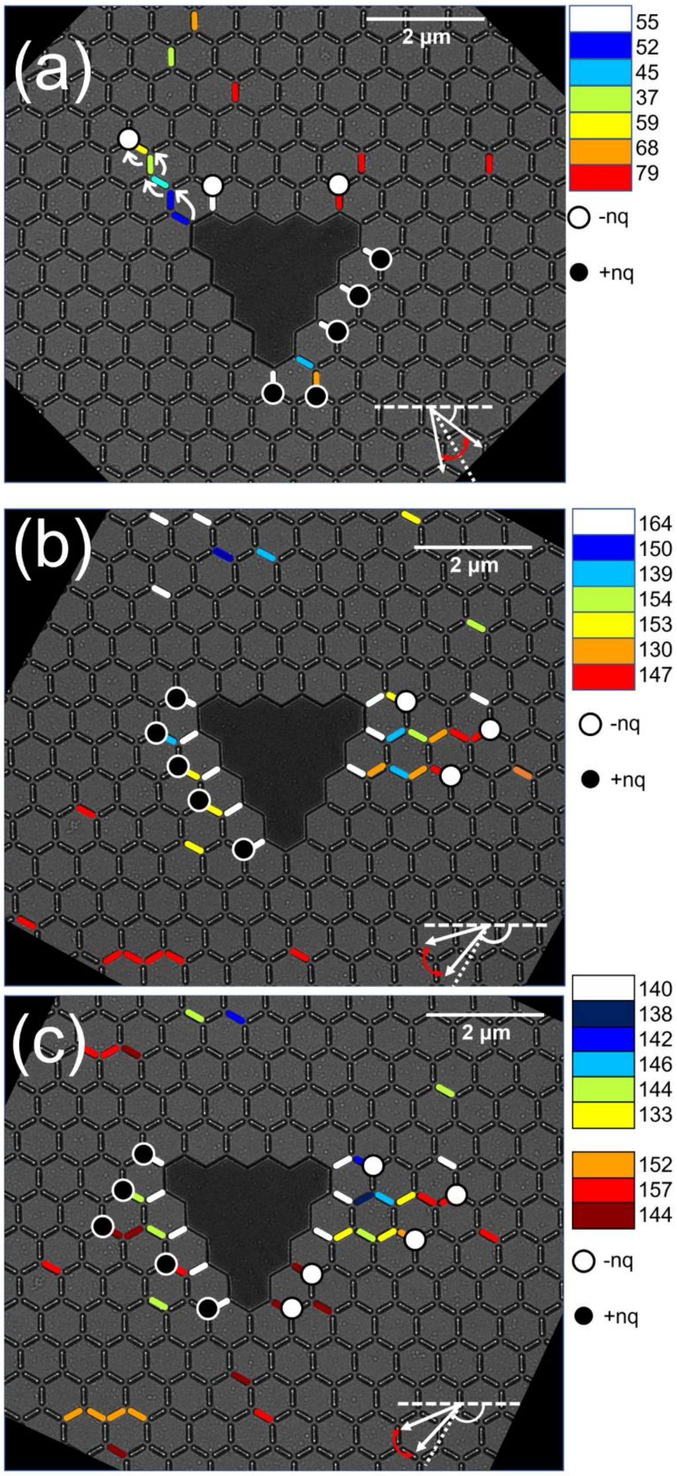

Supplementary Fig. 13. *LTEM images of Defect ASI lattice as it undergoes the field protocol described by the color scale ($\mu_0 H_{||}$= 21 mT). Colored islands represent NI switching relative to the color-bar provided, and final positions of defect-bound negative (white) and positive (black) MPs are indicated by circles. Solid and dashed white arrows indicate one-step and two-step MP propagation mechanisms, respectively; and dashed red-line indicates the closest zigzag axis to the net field direction (bottom-right of each image).*

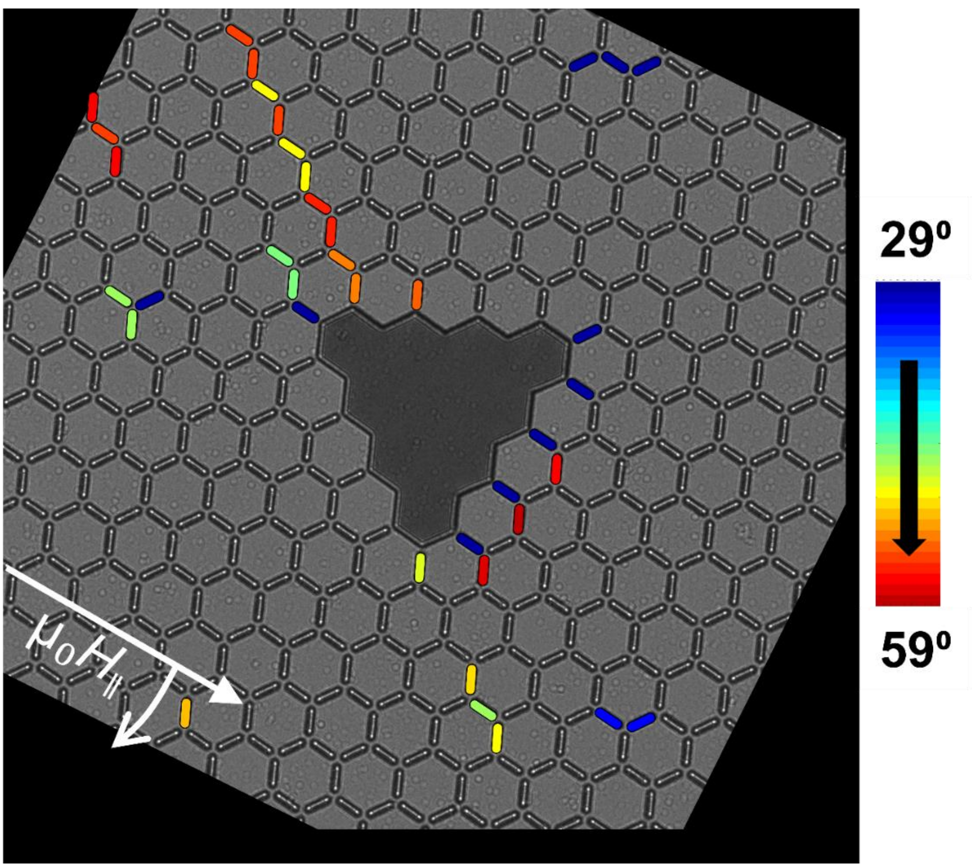

Supplementary Fig. 14. *LTEM image of DASI lattice as it undergoes coherent rotation of field, $\mu_o H_{||}$= 21 mT, from armchair to zigzag axis alignment (29° - 59°), where colored islands are relative to the color-bar.*

Supplementary References

[1] E. Mengotti *et al.*, *Nat. Phys.* **2011**, *7*, 68.

[2] A. Farhan *et al.*, *Phys. Rev. B* **2017**, *96*, 064409.

[3] J. P. Morgan *et al.*, *New J. Phys.* **2011**, *13*.
