# Supplementary material for: Defect-induced monopole injection and manipulation in artificial spin ice
Source: Nat Commun. 2022 Jun 25;13:3641. doi: 10.1038/s41467-022-31309-0 (PMC9233697; doi:10.1038/s41467-022-31309-0)
Supplement: Supplementary file 2 — Description of Additional Supplementary files [file 41467_2022_31309_MOESM2_ESM.docx]

# Description of Additional Supplementary Files: Defect-induced monopole injection and manipulation in artificial spin ice

R. Puttock *et al.*

File Name: Supplementary Movie 1

Description: Raw Lorentz TEM images of DASI undergoing magnetization reversal as the field is applied along the armchair axis (towards the top left of the images).

File Name: Supplementary Movie 2

Description: Raw Lorentz TEM images of DASI undergoing magnetization reversal as the field is applied along the armchair axis (towards the bottom right of the images).

File Name: Supplementary Movie 3

Description: Raw Lorentz TEM images of DASI undergoing magnetization reversal as the field is applied along the zigzag axis (towards the top left of the images).

File Name: Supplementary Movie 4

Description: Raw Lorentz TEM images of DASI undergoing magnetization reversal as the field is applied along the zigzag axis (towards the bottom right of the images).

File Name: Supplementary Movie 5

Description: Raw Lorentz TEM images of vacant DASI undergoing magnetization reversal as the field is applied along the armchair axis (towards the top left of the images).

File Name: Supplementary Movie 6

Description: Raw Lorentz TEM images of vacant DASI undergoing magnetization reversal as the field is applied along the armchair axis (towards the bottom right of the images).

File Name: Supplementary Movie 7

Description: GIF of the 2D planar field magnitude colormaps of the modelled full small DASI lattice as it undergoes magnetisation reversal. Arrows represent the discretized magnetization vector.
