# Supplementary figures and images for: Defect-induced monopole injection and manipulation in artificial spin ice
Source: Nat Commun. 2022 Jun 25;13:3641. doi: 10.1038/s41467-022-31309-0 (PMC9233697; doi:10.1038/s41467-022-31309-0)

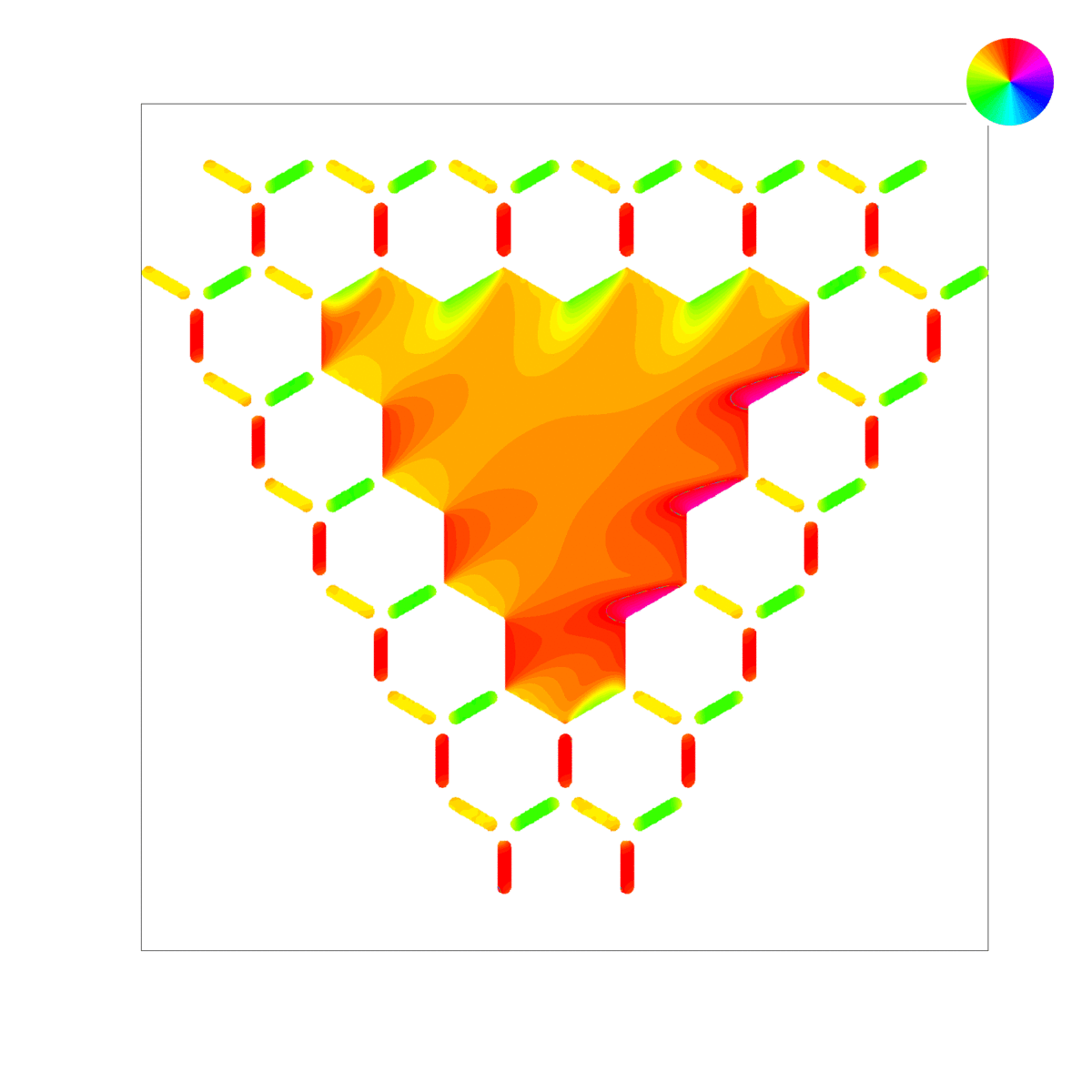

Supplement: Supplementary file 9 — Supplementary Movie 7 [file 41467_2022_31309_MOESM9_ESM.gif]
